# Ku Binding on Telomeres Occurs at Sites Distal from the Physical Chromosome Ends

**DOI:** 10.1371/journal.pgen.1006479

**Published:** 2016-12-08

**Authors:** Mélanie V. Larcher, Emeline Pasquier, R. Stephen MacDonald, Raymund J. Wellinger

**Affiliations:** Department of Microbiology and Infectious Diseases, Faculty of Medicine and Health Sciences, Université de Sherbrooke, Sherbrooke, Canada; Chinese Academy of Sciences, CHINA

## Abstract

The Ku complex binds non-specifically to DNA breaks and ensures repair via NHEJ. However, Ku is also known to bind directly to telomeric DNA ends and its presence there is associated with telomere capping, but avoiding NHEJ. How the complex discriminates between a DNA break and a telomeric extremity remains unknown. Our results using a tagged Ku complex, or a chromosome end capturing method, in budding yeast show that yKu association with telomeres can occur at sites distant from the physical end, on sub-telomeric elements, as well as on interstitial telomeric repeats. Consistent with previous studies, our results also show that yKu associates with telomeres in two distinct and independent ways: either via protein-protein interactions between Yku80 and Sir4 or via direct DNA binding. Importantly, yKu associates with the new sites reported here via both modes. Therefore, in *sir4Δ* cells, telomere bound yKu molecules must have loaded from a DNA-end near the transition of non-telomeric to telomeric repeat sequences. Such ends may have been one sided DNA breaks that occur as a consequence of stalled replication forks on or near telomeric repeat DNA. Altogether, the results predict a new model for yKu function at telomeres that involves yKu binding at one-sided DNA breaks caused by replication stalling. On telomere proximal chromatin, this binding is not followed by initiation of non-homologous end-joining, but rather by break-induced replication or repeat elongation by telomerase. After repair, the yKu-distal portion of telomeres is bound by Rap1, which in turn reduces the potential for yKu to mediate NHEJ. These results thus propose a solution to a long-standing conundrum, namely how to accommodate the apparently conflicting functions of Ku on telomeres.

## Author Summary

The Ku complex binds to and mediates the rejoining of two DNA ends that were generated by a double-stranded DNA break in the genome. However, Ku is known to be present at telomeres as well. If it would induce end-to-end joining there, it would create chromosome end-fusions that inevitably will lead to gross chromosome rearrangements and genome instability, common hallmarks for cancer initiation. Our results here show that Ku actually is associated with sites on telomeric regions that are distant from the physical ends of the chromosomes. We propose that this association serves to rescue DNA replication that has difficulty passing through telomeric chromatin. If so called one-sided breaks occur near or in telomeric repeats, they will generate critically short telomeres that need to be elongated. The binding of Ku may thus either facilitate the establishment of a specialized end-copying mechanism, called break induced replication or aid in recruiting telomerase to the short ends. These findings thus propose ways to potential solutions for the major conceptual problem that arose with the finding that Ku is associated with telomeres.

## Introduction

The Ku proteins, initially identified as an auto-antigen in sera from patients suffering of scleroderma-polymyositis overlap syndrome [1], are highly conserved in eukaryotes and there are also prokaryotic equivalents [2]. In eukaryotes, two subunits, Ku70 and Ku80, form a complex and its crystal structure revealed resemblances to a preformed ring [3]. This Ku-complex selectively associates with ends of double-stranded DNA molecules with high affinity but no sequence specificity [2, 4]. Ku’s primary function is to mediate Non-Homologous End Joining (NHEJ), the predominant DNA double-strand break (DSB) repair mechanism in mammals [4, 5]. However and paradoxically, in many species Ku does associate with telomeres and/or telomerase and a number of telomere-specific functions for Ku have been described [4]. How these telomere-specific functions that are thought to preclude DNA-end fusions discriminate telomeres from DSBs, where DNA-end fusions are the desired outcome, remains unknown.

The budding yeast *S*. *cerevisiae* also contains a yKu complex formed by Yku70 and Yku80 subunits [6–8]. As in mammals, yKu is essential for NHEJ, but not for Homologous Recombination (HR) [7]. yKu binds telomeres [9] and once there, supports functions such as inhibition of 5’-end resection [9, 10], telomere position effect (TPE) [9, 11, 12], and intranuclear positioning of telomeres [13]. Moreover, yKu, by its interaction with the RNA component of telomerase, is important for telomeric DNA maintenance and nuclear localization of telomerase [14, 15]. While it is clear that in principle, yKu can directly bind at an end of double stranded telomeric DNA as well as a stem-loop structure on the RNA component of telomerase, most likely those interactions occur on the same interface on yKu, and therefore are mutually exclusive [16]. Moreover, there is evidence that Yku80 interacts with Sir4 [17, 18], and at least some yKu complexes may associate with telomeres via this indirect protein-protein interaction [16, 19].

**Fig 1:**
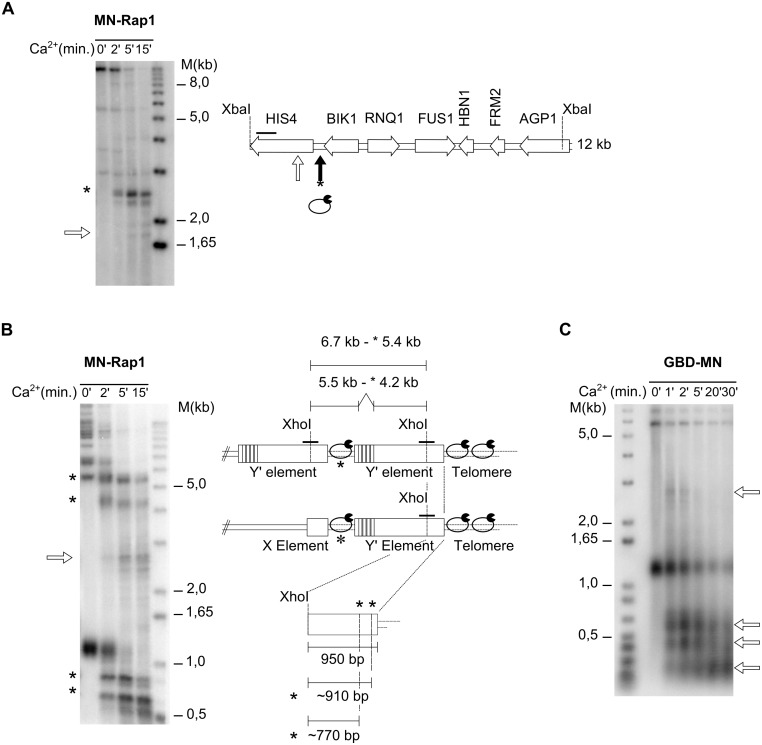
Rap1 binding detected using a MN-Rap1 fusion protein. **A** *In vivo* ChEC with MN-Rap1 analyzed on the non-telomeric *HIS4* region on chromosome III. Left; Southern blot with XbaI digested genomic DNA. Time of MNase induction by the addition of Ca^2+^ is indicated on top of the gel. Ca^2+^ induced cutting is indicated by a * and weak, non-specific cutting by open arrow. The analyzed locus is schematized on right with the known Rap1-binding site indicated by black arrow. **B** Same analysis as in A, but on a chromosome terminal region using a Y’-specific probe. The genomic DNA was digested with XhoI and the terminal region is drawn with an X-element and either one or two Y’-elements in tandem (right). Note that there are telomeric repeats between X and the Y’ elements as well as between the tandem Y’-elements. Ca^2+^-induced cutting will shorten Y’-elements to about 5.4 kb for Y’-long and to 4.2 kb for Y’-short, indicated by * top part of gel). The fragments generated on the terminal restriction fragment are also indicated by *. Non-specific cutting at 2.7 kb is indicated by a white arrow. **C** ChEC *in vivo* with a GBD-MN fusion protein and analyzing the same chromosome terminal region as in B. The GBD-MN protein is overexpressed on a plasmid and there are no Gal4-binding sequences in the analyzed area. MNase cutting indicates MNase accessible sites after Ca^2+^ induction.

As mentioned above, the differentiation of Ku-binding at DSBs which is instrumental for NHEJ and the binding mode on telomeres, where end-fusions must be avoided, is unknown. Previous results suggested a “two faces” idea for yKu’s association with DNA-ends [20]. In this model, most of the Yku80 side faces inward from the end and is essential for yKu’s telomeric functions. Yku70, facing towards the end, would be essential for yKu’s role in NHEJ [20]. Telomeric DNA is particular and composed of tandem repeats of G-rich sequences [21]. Budding yeast telomeric repeat DNA is 300 bp +/- 75 bp long (commonly abbreviated (C_1-3_ A)_n_−(TG_1-3_)_n_) and a number of proteins are associated with these repeats: Rap1 binds directly and with high affinity to a consensus sequence in the repeats, and Rif1 and Rif2 as well as the Sir2/Sir3/Sir4 proteins associate with telomeres via Rap1 [21]. Eventually, it is the resulting nucleoproteic structure that ensures the functions ascribed to telomeres [21]. However, in addition to their localization at chromosomal termini, in many eukaryotic species telomeric repeats are also present at internal genomic sites and have been dubbed interstitial telomeric sequences (ITSs) [22, 23]. In yeast sub-telomeric regions, ITSs are relatively frequent and they are thought to set the boundaries between different telomere-associated elements [24]. These elements include heterogeneous X elements that are found on all telomeres, with sizes varying between 0.3 kb to 3.7 kb [24–26]. Y’ elements, unlike the X elements, are found on about half of the telomeres, are much more homogeneous, and occur in two size classes, ~ 5.5 kb (Y’ short) and ~ 6.7 kb (Y’ long). Y’-elements can occur in tandem with 1 to 4 copies and, if present, they are positioned immediately next to the terminal repeats [25, 26]. The ITSs between these sub-telomeric elements vary between 50 to 150 bps (Fig 1, [24]). Importantly, telomeric repeats at chromosome ends and at ITSs are well characterized natural replication barriers, causing replication forks to stall at those sites [27–30]. Furthermore, there is direct evidence that such stalled or stressed replication forks are converted to DNA double-strand breaks (DSBs) [31]. For mammals, a very close association of ITSs with chromosome breakage has been documented [32, 33], and if not repaired adequately, these breaks will compromise genome stability and cell viability [34, 35].

In order to investigate how yKu can be bound at telomeres, and at the same time be prevented from mediating NHEJ, we used *in vivo* Chromatin Endogenous Cleavage (ChEC; [36]) coupled to Southern blots to pinpoint yKu’s localization. The results show that the yKu complex is found associated with telomeric repeats in or near ITSs and on terminal repeats that are distal from the physical ends of chromosomes. Consistent with previous results, a fraction of this internal yKu association is dependent on Sir4, but there clearly is also Sir4 independent binding. Remarkably, telomeric yKu can be trapped on an inducibly excised circular DNA molecule with telomeric repeats, but not if there are no telomeric repeats on it. Furthermore, by using an inducibly tagged yKu, the results show that new associations of yKu with ITSs are dependent on the passage through S-phase. These observations lead us to propose that on telomeres, yKu may be bound predominantly on internal repeat sites, allowing for the presence of telomeric chromatin in the portion of the telomeric repeats that is distal to yKu. This would occlude the Yku70-NHEJ side from the physical ends and explain why yKu binding at telomeres is very important for telomere integrity, while at the same time incapable of engaging NHEJ.

## Results

### Application of *in vivo* ChEC on telomeric proteins

The ChEC method was developed in order to map the binding sites of proteins within their endogenous chromatin landscape [36]. The method is based on cleavage of native chromatin by Miccrococal Nuclease (MN) that is fused to proteins of interest. The actual DNA cleavage is induced by external addition of calcium, the concentration of which in a yeast nucleus normally is too low to activate the MN. Determination of actual cleavage sites is done by Southern-blotting (S1A Fig). Here, we intended to pinpoint positions of the yKu-complex on genomic loci of *S*. *cerevisiae*. As a control, we first constructed MN-Rap1, which had already been shown to be amenable to this technique [37]. Yeast telomeric repeats contain the highest affinity sites for Rap1 and on average, there are 15–20 Rap1 proteins on each yeast telomere [38]. However, the protein also recognizes sites in many transcriptional promoters [37, 39]. For a first assessment of *in vivo* ChEC, we performed experiments with MN-Rap1 and analyzed the *HIS4* locus with one Rap1 binding site (Fig 1A, S1B Fig), the *RPL21a* locus with two sites (S1C Fig) and telomeres with many sites (Fig 1B). For the *HIS4* locus, before Ca^2+^ addition, the XbaI restriction fragment detected is at ~ 12.0 kb, as expected (Fig 1A, S1B Fig). Within 2 min after Ca^2+^ addition, a new fragment of about 2.5 kb (*) was detectable. This fragment corresponds to a cleavage at the expected Rap1 binding site and progressively becomes the major fragment. At later time points, low-intensity fragments are also generated (white arrow in Fig 1A) and those correspond to MN-hypersensitive sites without specific Rap1 binding. Such a two tiered appearance of sites (early with specific Rap1 binding and later without Rap1 binding) is consistent with a previous report on ChEC with Rap1 [37]. Quite analogous results were obtained when the RPL21a locus with two Rap1 binding sites was analyzed (S1C Fig). Finally, MN-Rap1 binding at telomeres caused a fast disappearance of the terminal restriction fragment and the appearance of two new bands at ~ 910 bp and ~ 770 bp (Fig 1B). Of note, on the Y’-elements, about 950 bp separate the XhoI restriction site from the beginning of the terminal repeats, suggesting that the detected major cleavage via induced MN-Rap1 occurred near the transition between Y’ and terminal repeats (Fig 1B). In addition and as expected, MN-Rap1 also mediated cleavage in the ITS loci. Because the Y’-specific probe used here covers sequences on both sides of the XhoI site in the Y’-element, the detected internal Y’-fragments (either a full Y’-element with the ITS in case of a tandem Y’, or the X-ITS-Y’ fragments, see drawing in Fig 1B) were shortened to yield ITS-XhoI fragments (Fig 1B, * near 4.2 and 5.4 kb). As described before [37], longer induction of MN-Rap1 cleavage also yielded some non-specific fragmentation (see empty arrow, about 2.5 kb in Fig 1B). In order to discriminate between such non-specific cleavage sites and those induced by Rap1 binding to cognate sites, we compared the MN-Rap1 cleavage pattern with that produced with a GBD-MN, where MN is fused to the Gal4 DNA binding domain (Fig 1C). GBD-MN also created the non-specific 2.5 kb fragment and a number of new fragments that could correspond to nucleosomal arrays near the probe, i.e. generating very small sized fragments at the bottom of the gel. In contrast to when MN-Rap1 was used however, with GBD-MN we did not observe cleavage at ITS sites or at the sub-telomere-telomere junctions (Fig 1C). These results indicate that at yeast telomeres, MN-Rap1 does indeed induce specific cleavages within 100–200 bp of its binding sites on terminal and ITS telomeric repeats.

### yKu localizes internally on terminal telomeric repeats tracts and on ITSs

**Fig 2:**
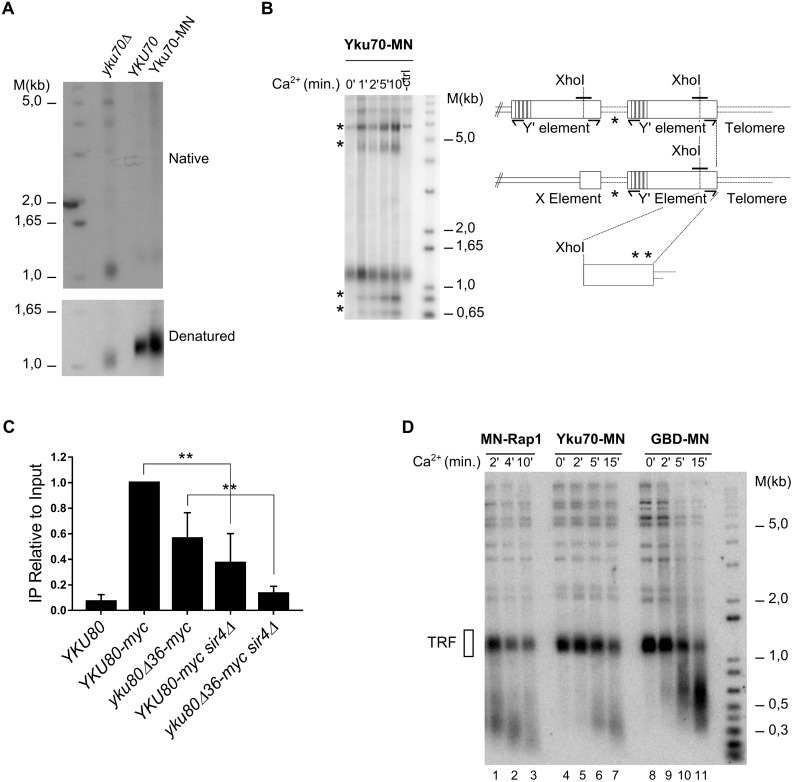
yKu localization on telomeric DNA. **A** Non denaturing in-gel hybridization using a CA oligonucleotide probe with XhoI-digested DNA derived from cells with the indicated *YKU70* alleles (top). To control the DNA loading, DNA was denatured, transferred to a nitrocellulose membrane and hybridized with a telomeric probe (bottom). Note that the absence of yKu (*yku70Δ* allele) causes very short telomeres with a constitutive G-strand overhang. **B** ChEC *in vivo* with the Yku70-MN protein and DNA analysis as in Fig 1B. After Ca^2+^ addition, new fragments (marked with *) are detectable. Measured fragment sizes were 5.4, 4.2, 0.91 and 0.77 kb. Schematic of telomeric areas probed. XhoI sites are indicated and the short solid black line shows the probe. **C** ChIP analysis with Myc-tagged Yku80 proteins and primer pairs specific for the ITS loci on Chr XII indicated (see B). Yku80Δ36 lost its ability to bind nucleic acids. P values were calculated by the unpaired t-test with Welch’s correction considering significance of differences are noted as follows: P> 0,05; * P≤ 0,05; ** P≤ 0,01; *** P≤ 0,001. At least three independent biological experiments were performed for each case. **D** Same assays as in Fig 2B, but the blot was hybridized with a telomeric repeat specific probe. MN-fusion proteins used are indicated on top of the gels.

The precise location of the yKu complex on telomeres still is unclear. We thus wished to determine those sites using the above described *in vivo* ChEC method. The Yku70 protein was tagged with MN, creating Yku70-MN, and we analyzed telomeric cleavages by southern blot analyses as above. Without Ca^2+^ addition, the detected terminal restriction fragment (TRF) pattern of the strain was indistinguishable from a wild-type strain and we did not observe any increase in telomeric overhang signal, indicating that the fusion of MN to Yku70 does not impinge on yKu-function (Fig 2A). Upon MN induction, a very comparable TRF pattern as the one obtained for MN-Rap1 is observed: Yku70-MN cleavage generated 910 bp and 770 bp fragments, corresponding to a cleavage at the subtelomere-telomere junction and one about 140 bp distal to that junction. Remarkably, Yku70-MN cleavage was also detected near or on the ITS sequences between the subtelomeric repeats: the same two ITS-XhoI fragments as for the MN-Rap1 cleavage are detected in the upper area of the gel (* in Fig 2B). Previous studies already suggested an association of yKu with sequences in or near subtelomeric X-elements, which may have reflected yKu association with ITSs [40]. In order to confirm the yKu association with ITSs without the complication of a nearby X-element, we performed an experimentally independent approach to assess this yKu-ITS association. We chose to use chromatin immunoprecipitation (ChIP) using Myc-tagged Yku80 followed by q-PCR using primers that are specific for ITSs that occur between two Y’-elements on chromosome 12 (TelXIIL and TelXIIR; Fig 2B and 2C). As the ChEC results above suggested, these ITS loci are indeed efficiently immunoprecipitated when the Yku80 protein is tagged, but not if an untagged construct is used (Fig 2C, left). Furthermore, as will be shown below, yKu also associates with artificial ITSs on linear plasmids and ITSs that are far from the next telomeric region. Finally, DNA samples derived from ChEC analyses with MN-Rap1, Yku70-MN or GBD-MN were also analyzed by probing with a telomere repeat specific probe (Fig 2D). Consistent with the idea that Rap1 binds throughout on telomeric tracts, after 10 min of induction, ChEC with MN-Rap1 creates very short DNA fragments of less than 250 bp (Fig 1D, lane 3). In contrast, Yku70-MN induced cutting creates telomeric repeat containing fragments that seem to plateau at around 350 bp, even after 15 min of ChEC induction (Fig 1D, lane 7). The specificity of those cuts is underscored by the fact that ChEC with GBD-MN creates an entirely different pattern (Fig 1D, lanes 8–12), creating much larger fragments of over 600 bp that could correspond to what was called the telosome previously [41]. Collectively, these data confirm that yKu is specifically associated with telomeric repeat tracts. However, as opposed to what is expected from its end-binding property, on telomeres the yKu complex appears associated with repeats near the telomere-subtelomere junction and on telomeric ITSs.

### Sir4 independent binding of yKu on telomeric repeat DNA

**Fig 3:**
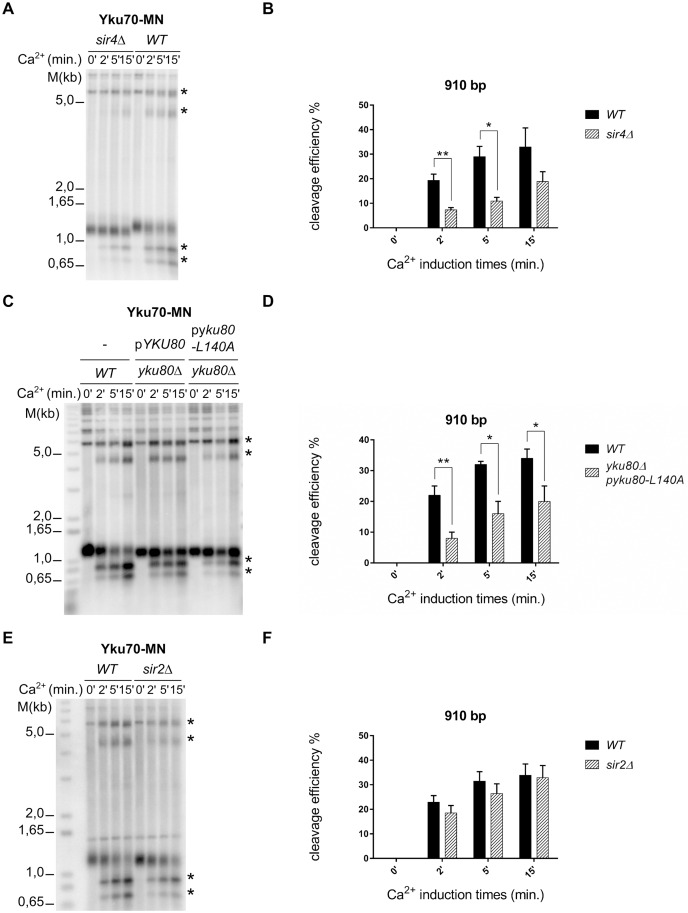
yKu-association with telomeric repeats is only partially Sir4-dependent. **A** *In vivo* ChEC with Yku70-MN was performed and analyzed as in Fig 2. Strains were either *SIR4* (WT, right part) or contained a *sir4Δ* allele (left part of gel). Ca^2+^ induction and dependent fragments are indicated as in Figs 1 and 2. **B** Quantification of the generation of the band at 910 bp in both strains with respect of Ca^2+^ addition. Quantification was realized on three independent experiments. Differences between the WT and mutant strains (P-values) were calculated as for Fig 2C. **C** Same as in A except that Yku80-L140A was used. **D** Quantification of C as in B. **E, F** Same as in A, B, except that a strain harbouring a *sir2Δ* allele was used.

Given this presence of yKu on sites relatively distant from the actual chromosome terminus and on ITSs, we wondered whether the reason for this association was direct DNA-binding or a possible indirect association. yKu is known to interact with Sir4 via the Yku80 subunit and there is previous evidence for two pools of yKu on telomeres: one that is bound directly on DNA and one that is associated indirectly via this Sir4-Yku80 interaction [17, 19, 20]. Moreover, there are *YKU80* separation-of-function (SoF) alleles, which display a drastically reduced interaction with Sir4 and are dysfunctional in telomeric gene silencing, but are proficient in NHEJ and telomeric repeat DNA maintenance [17, 20]. These alleles thus are thought to be fully proficient in DNA binding. Finally, on telomeric DNA, Rap1 association with only Sir4 is sufficient to trigger the establishment of a specialized telomeric chromatin [42]. In order to assess a possible Sir4-dependence of the yKu-telomere interactions detected in our assays, we combined a *sir4Δ* allele or a *YKU80* SoF allele with the *yku70-MN* allele and performed *in vivo* ChEC on these strains. Qualitatively, the Yku70-MN-mediated cleavage profile on telomeres is very similar in *SIR4* and in *sir4Δ* cells (Fig 3A). After Ca^2+^ addition, the same two terminal fragments of 910 bp and 770 bp are generated as in the WT cells and the subtelomeric elements are also cleaved in the ITSs that separate them. However, cleavage efficiency at the different sites was reproducibly reduced in *sir4Δ* cells as compared to WT, in particular at early time points of MN induction (Fig 3B and S2A Fig). For example, 2 min after Ca^2+^ addition, Yku70-MN cleavage efficiency in the *sir4Δ* cells is 2–3 fold lower for both the 910 bp and 770 bp fragments as compared to the efficiencies observed in wild type cells (WT_910_: 19,37%; *sir4Δ*_910_: 9,39%; WT_770_: 9,79%; *sir4Δ*_770_: 3,70%).

The Yku80 α-helix 5 is essential for telomeric silencing and Sir4 binding [20]. Specifically, cells harbouring the *yku80-L140A* allele present a silencing defect and reduced Yku80-Sir4 interaction, but telomere length and NHEJ are not affected. Thus, in order for an independent assessment of the observations made with *sir4Δ* cells, we tested Yku70-MN ChEC cleavage in cells with this *yku80-L140A* allele. We used two strains derived from the Yku70-MN strain, both harbouring a *yku80Δ* allele at the genomic locus. yKu function was then re-established via plasmid borne expression of wild type Yku80 from its endogenous promoter or a plasmid borne expression of *yku80-L140A*. As was observed in the *sir4Δ* strains, the cleavage profile qualitatively was not affected in cells expressing the Yku80-L140A protein (Fig 3C). Moreover, cleavage efficiencies were similarly reduced as in the *sir4Δ* strains (Fig 3D and S2B Fig). These findings with the ChEC technique were confirmed by ChIP with q-PCR: immunoprecipitation of ITS loci in *sir4Δ* cells was significantly reduced when compared to *SIR4* WT cells (Fig 2C). In these ChIP experiments, we also used a strain expressing a Myc-tagged Yku80Δ36 protein, which is unable to bind any nucleic acid (either DNA or RNA)[16]. The immunoprecipitates with this protein did still contain ITS loci, albeit in reduced amounts when compared to the amounts detected with wt tagged Yku80 (Fig 2C). Finally, when we expressed the Myc-tagged Yku80Δ36 protein in *sir4Δ* cells, the ChIP signals were reduced to background levels. These results show that yKu associates with internal telomeric repeats in two ways: either by direct DNA binding or via an indirect Sir4-mediated association. The reduced ChEC cleavage in *sir4Δ* cells above therefore is due to a reduced presence of yKu on telomeric repeats, but the yKu-complexes still remaining are directly bound on the very same sites within telomeric repeat DNA.

It could be argued that the reduced cleavage reported above was due to an altered chromatin configuration at telomeres, but not due to a loss of the specific Yku80-Sir4 interaction. In order to investigate this possibility, we performed Yku70-MN-mediated ChEC in a strain harbouring a *sir2Δ* allele. Sir2 is a conserved NAD+ dependent histone deacetylase [43–45] that, together with the Sir3 and Sir4 proteins, is required for the specialized chromatin at telomeres [21, 42, 46]. The cleavage profile in *sir2Δ* cells again is similar to that observed in wild type cells (Fig 3E). However, as opposed to what was observed in the *sir4Δ* strains, cleavage efficiencies for the 910 bp and 770 bp fragments only decreased marginally and the decrease for the most part was not statistically significant (Fig 3F and S2C Fig). Furthermore, we also analyzed MN-Rap1 mediated cleavage in *sir4Δ* cells. As expected, there were no qualitative or quantitative differences in the cleavage patterns observed between *SIR4* and *sir4Δ* cells (S3A and S3B Fig).

Altogether, these observations are in line with previous results that suggested that the Yku80-Sir4 interaction is important for yKu-mediated roles in chromatin related functions, but not for direct binding of yKu on telomeric DNA [47, 48]. Hence, the Sir4-independent Yku70-MN mediated cleavages we detect on telomeric chromatin are due to yKu being bound on DNA.

### yKu is associated with an excised telomeric repeat tract

**Fig 4:**
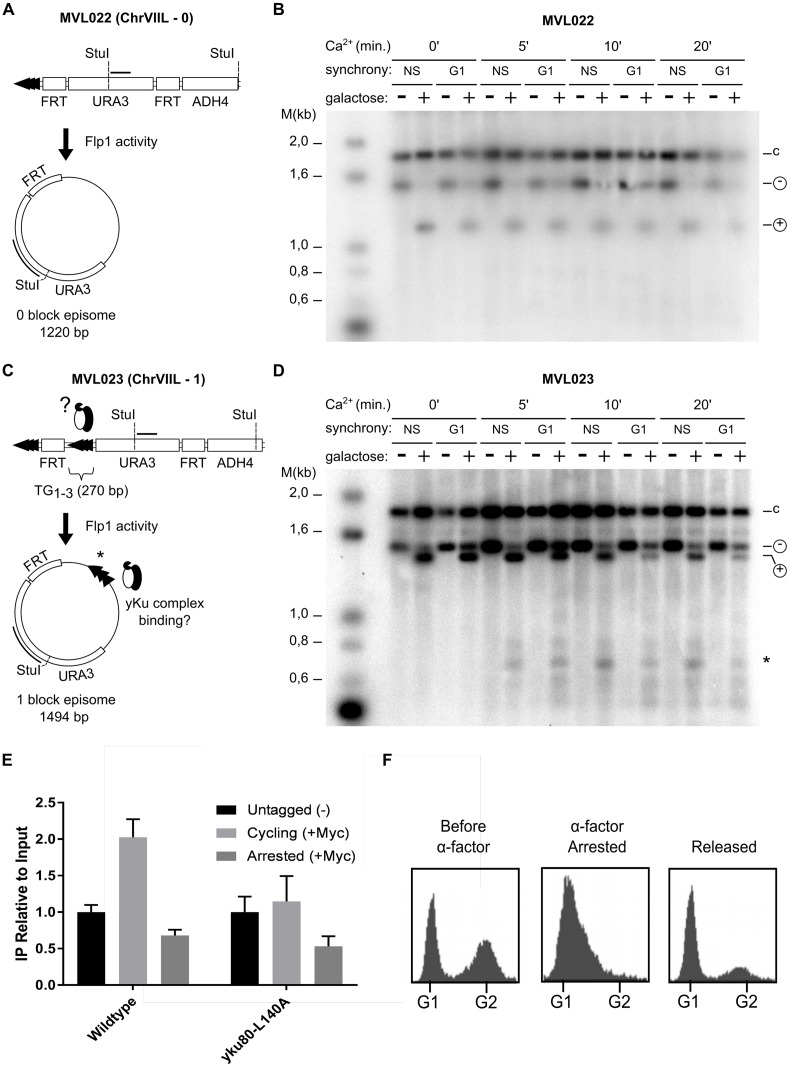
yKu is associated with internal telomeric repeats. **A** and **C:** schematics of the modified chromosome VIIL structures in the strains used. Note that ChrVIIL-0 has no telomeric repeats between the two FRT sites, whereas the ChrVIIL-1 contains one block of 270 bp telomeric repeats. After induction of FLP-activity, the circular plasmids as indicated below are generated. **B** and **D** Southern blots of DNAs isolated from strains harboring the Yku70-MN fusion protein and either of the two chromosome VIIL constructs. Induction of MNase by Ca^2+^ is indicated on top of the gel (0 means DNA isolated before addition of Ca^2+^). Induction of FLP recombinase is indicated by:—: cells grown in raffinose; +: cells grown in galactose. NS: cells were in exponential growth phase; G1: cells were arrested in G1 phase of the cell cycle. Total DNA was digested with StuI and the blot was hybridized to a probe specific for a part of the *URA3* gene as indicated on the episome. Symbols on the right of the gels indicate: c: chromosomal *URA3* locus (1.87 kb); -: StuI fragment on chromosome VIIL before FLP induction (1.56 kb); +: StuI linearized episome after FLP induction (either 1.22 kb or 1.49 kb). *: 0.75 kb episomal fragment generated by Yku70-MNase. **E** ChIP efficiency as assessed by qPCR of induced Yku80-myc or Yku80-L140A-myc constructs on ITS loci on Chr XII (Fig 2B) in G1 (“Arrested”) or growing cells (“Cycling”). See S4 Fig for details of the experimental setup. Yku80-L140A lost its ability to interact with Sir4. IPs were normalized relative to their respective inputs. Untagged (before galactose induction) values were normalized as 1. **F** FACS analyses of the cultures harbouring the *YKU80* wt allele as shown in E.

The above observations predict that at least part of yKu was in fact not bound at the very ends of chromosomes, but rather at internal sites of telomeric repeat tracts. In order to verify this prediction, we used a telomeric repeat flip-out system that should trap internally bound yKu on a circular DNA molecule, while yKu associated with the distal-most part of the telomere would remain on the chromosome, even after flip-out (see Fig 4 and [49]). We thus constructed strains in which the extremity of chromosome VIIL is modified accordingly and that also contained the *yku70-MN* allele. In addition, the strains contained a copy of Pgal10*-FLP1* integrated in the *LEU2* locus on chromosome III, which allows for a galactose-inducible Flp1-recombinase expression. The first strain, MVL022, has the *URA3* gene flanked by the two Flp1-recognition target sites (FRT) (ChrVIIL-0 block, see Fig 4A) and the second strain, MVL023, has an additional TG_1-3_ telomeric tract of 270 bp between the first FRT site and the *URA3* marker gene (ChrVIIL-1 block, Fig 4C). Flp1 recombinase induction by addition of galactose causes recombination between the repeated FRT sites and all sequences between the FRT sites will end up on an excised circular DNA molecule. In MVL022, this circular molecule will contain the *URA3* marker and an FRT site, while in MVL023, it also contains the internal TG_1-3_ telomeric tract of the original telomere VIIL. Therefore, upon MN induction and digestion of the DNA with StuI, the latter linearized fragment with be further cut only if yKu was associated with internal telomeric repeats, but not, if it was localized exclusively in the most distal portion of the telomere. Both strains MVL022 and MVL023 were incubated in media containing 2% galactose to induce Flp1 expression or kept in 2% raffinose as non-induced controls. In addition, part of the cultures was maintained in G1 by adding α-factor for 1.5 hours prior to Flp1 induction. *In vivo* ChEC was performed on all strains, followed by DNA analyses on southern blots (Fig 4B and 4D). In both strains and all conditions, the *URA3* probe detects a fragment at 1875 bp which corresponds to the StuI fragment from the endogenous genomic *URA3* locus (-c in Fig 4B and 4D). For strain MVL022, when Flp1 is not induced, the fragment at 1560 bp corresponds to the restriction fragment between the two StuI sites on the modified ChrVIIL (marked with Θ, Fig 4B). After galactose addition, a new fragment appears at 1220 bp corresponding to the StuI linearized form of the circular molecule (marked with +, Fig 4B). Addition of Ca^2+^ and induction of the MN did not change this pattern, even after 20 min of induction. This suggests that in the absence of telomeric repeats, the yKu complex does not associate with sequences in between the two FRT sites on the modified ChrVIIL. For strain MVL023 that contains a block of 270 bp telomeric repeats between the FRT sites, a fragment at 1494 bp corresponding to the StuI-linearized from of the circular molecule can be detected after Flp1-induction by galactose (marked +, Fig 4D). In addition and in stark contrast to strain MVL022, Ca^2+^ addition to these cells generates a new fragment at ~ 750 bp (see * in Fig 4D), which matches a predicted fragment, if Yku70-MN mediated cleavage occurred in or near the inserted TG_1-3_ repeat tract in the circular molecule. Given that a circular DNA molecule has no physical ends for yKu to bind to, we conclude that the yKu complex was already associated with the TG_1-3_ tract before circular molecule excision. In addition, we also performed this experiment in *sir4Δ* cells in order to exclude a protein mediated association of the excised circular DNA with telomeres. Consistent with the above Sir4-independent association of yKu with telomeric repeats, the FRT mediated recombined circular fragment with telomeric repeats is cleaved after ChEC induction and this cleavage is dependent on the presence of telomeric repeats (S3C Fig).

Unexpectedly, in the above assays, yKu was found to be bound on the excised circular DNA even if cells were arrested in G1, before excision of the circular DNA (see Fig 4D, lanes marked G1). In order to distinguish whether yKu could somehow associate with internal telomeric repeats during G1 or whether this yKu detection in G1 reflected trapped yKu from the last passage through the cell cycle, we mounted a system in which only new associations of yKu with telomeric repeat DNA are detected (see S4 Fig). In essence, the system is based on an inducible tagging of the Yku80 protein via site-directed recombination of two RS sites. This recombination is mediated by the bacterial RecR protein which in our case is expressed from the conditional gal promoter (S4A Fig). Thus, in cells grown with glucose or raffinose, there is a stop codon on *YKU80* ORF before the myc-peptides, the locus remains intact (S4B Fig) and no Yku80 is detected on a western blot probed with anti-myc antibodies (S4C Fig). However, 16 hrs after induction of the RecR protein by the addition of galactose to the media, most of the locus had recombined (S4B Fig) and Yku80-myc is now detectable on the western (S4C Fig). In addition to the wt *YKU80 ORF* on plasmid pEP22B, we also created the same taggable situation for the *yku80L140A* allele on plasmid pEP24C (S4D Fig). As a positive control for yKu association in the situations studied, cells also contained the plasmid YCpHOCut4, on which the HO-endonuclease is expressed from a galactose inducible promoter and an HO-cutting target sequence is integrated as well [50].The global cellular genetic make-up before the experiment is outlined in S4D Fig and the work-flow in S4E Fig. We thus assessed yKu binding after cells were arrested in G1 with α-factor, the myc-tagging of Yku80 induced by addition of galactose, followed by qChIP on ITS sequences on chromosome XII (see S4D Fig). If cells were allowed to grow after the *in vivo* tagging of Yku80, yKu could be found on ITSs as well as on the HO-cut plasmid, the positive control (lanes cycling + myc in Fig 4E and S4F Fig). However, if cells were retained in G1, the signal for yKu binding to ITSs remained as low as the untagged background (Fig 4E), even though the positive control clearly could be detected (S4F Fig, lane “Arrested +myc”). Arrest in G1 or release into the next cell cycle of the cell cultures was controlled by FACS analyses (Fig 4F). Similar results were also obtained with the *yku80L140A* allele (Fig 4E), even if the ITS binding by this protein during the next cycle was significantly lower than wt. Altogether, these results show that yKu is unable to associate with ITSs during G1 and that a passage through the next S-phase is required for this to happen.

### Replication fork direction does not influence yKu binding

**Fig 5:**
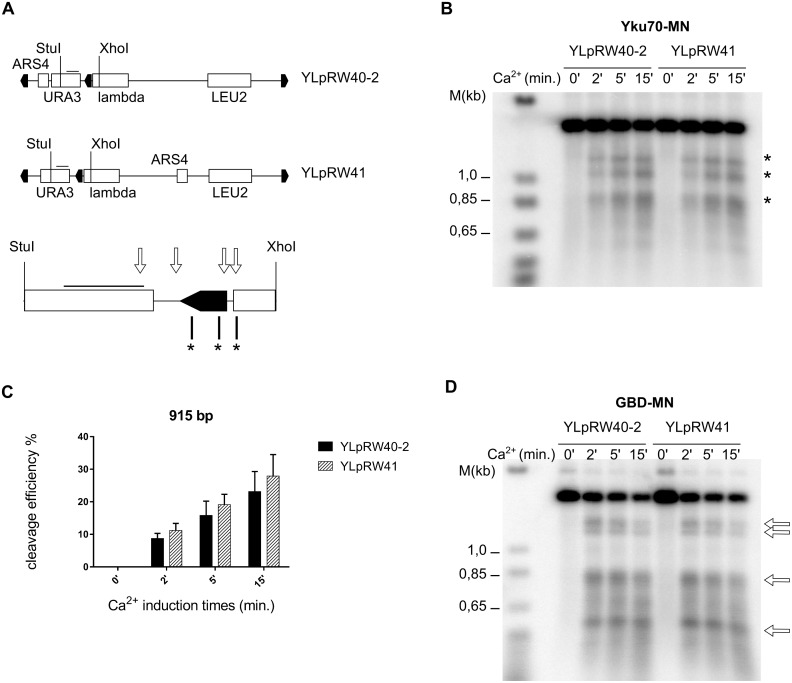
yKu-association with telomeric repeats is not influenced by the direction of fork passage. **A** Schematic drawings of the YLpRW40-2 and YLpRW41 plasmids with characteristic restriction enzyme sites and functional elements annotated. Below the plasmids, the analyzed StuI—XhoI is depicted. The three Ca^2+^ dependent cut sites marked in B are indicated below as *. GBD-MN cut sites are indicated by white arrows above. Note that due to the localization of the ARS element, the replication fork moves in opposite direction through the internal telomeric repeat tract. **B** *In vivo* ChEC with Yku70-MN in strains that harbour either YLpRW40-2 or YLpRW41. Total genomic DNA was digested with StuI and XhoI and the Southern blot was hybridized to a probe specific for *URA3*. Southern labeling and symbols are as before. **C** Quantification of the fragment arising at 915 bp on both plasmids with respect to MN-induction. P values were calculated as in Fig 2C and the values indicated no significant differences. **D** *In vivo* ChEC with a GBD-MN fusion protein and analyzing the same plasmid region as in B. The open white arrows indicate the non-specific sites as in A.

ITSs are known to be hot spots for the initiation of genomic rearrangements [32–35]. Previous results also reported replication fork stalling leading to double-strand breaks and chromosomal rearrangements due to telomeric repeat tracts [27, 28, 30]. We therefore surmised that the above results could be the consequence of DNA breaks occurring at ITSs during replication fork passage. In order to investigate this possibility, we analyzed yKu binding onto a specific and unique ITS engineered onto linear plasmids derived from plasmids YRpRW41 and YRpRW40-2 (Fig 5A; S4A Fig; [51]). The two linear constructs differed in the location of the origin of replication (Fig 5A) and hence, the directionality the replication fork is moving through the ITS. These plasmids were transformed into a strain with Yku70-MN, the MN induced by Ca^2+^ addition and the integrity of the 1.4 kb StuI-XhoI restriction fragment encompassing the ITS was analyzed by southern blotting (Fig 5B). The blots revealed three new fragments of 1160 bp, 1060 bp and 915 bp that were generated in a Ca^2+^ dependent fashion in both strains. All three sites map very close to, or within, the ITS tract, as indicated with * on Fig 5A. We also performed ChEC analysis with GBD-MN on these same plasmids and as expected did detect some non-specific sites that are cut by GBD-MN (Fig 5D, empty arrowheads). However, these non-specific sites mapped to quite distinct locations that differed from the Yku70-MN sites on the fragment (Fig 5A). Moreover, for both plasmids the cleavage profiles obtained with Yku70-MN are virtually the same and cleavage rates are also quite comparable with only a slight but not statistically different increase for YLpRW41 (Fig 5C, S4B Fig). These results are entirely consistent with previous physical studies that have mapped orientation-independent fork stalling due to ITSs [30]. This fork stalling thus may create one-sided breaks onto which yKu can load.

**Fig 6:**
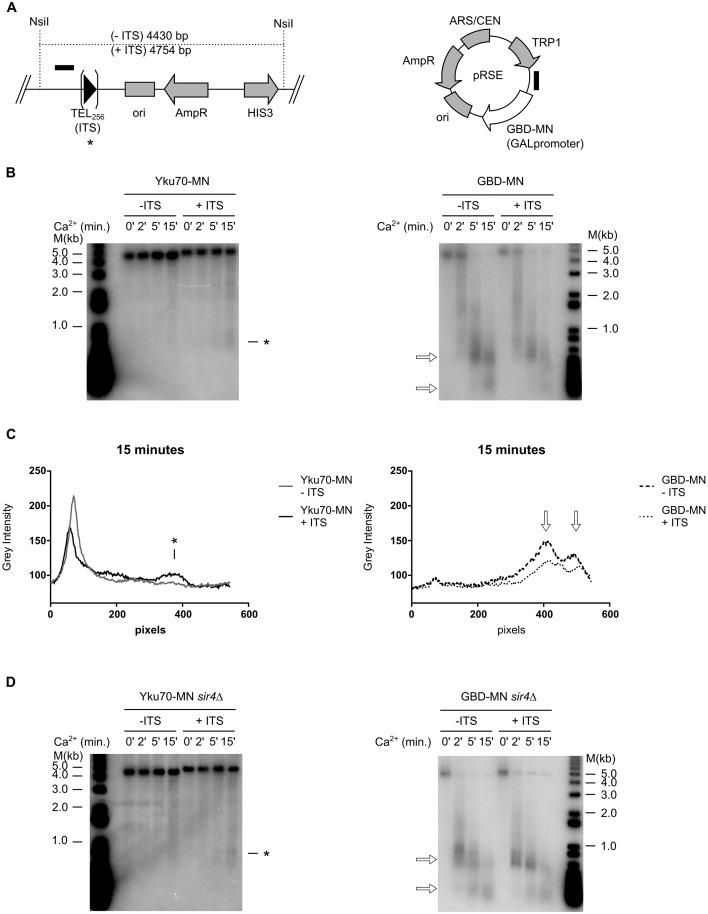
Cleavage by Yku70-MN on ITSs is not restricted to telomeric and subtelomeric regions. **A** (left) Schematic drawings of the modified *HIS3* locus with (+ITS) or without an inserted ITS (-ITS); and (right) of the pRSE plasmid that mediated galactose inducible expression of GBD-MN. Note that the probe used in B, indicated by a solid black line, hybridized with the locus of interest and the pRSE plasmid. **B** *In vivo* ChEC with Yku70-MN on the left and GBD-MN on the right analyzed on the *HIS3* loci depicted in A. Genomic DNA was digested with NsiI and the Southern Blot was hybridized with the plasmid specific probe indicated in A. Time of MNase induction by the addition of Ca^2+^ is indicated on top of the gel. Ca^2+^ induced cutting is indicated by a * for Yku70-MN, or by open arrows for non-specific cutting observed with GBD-MN as assessed after 45 minutes of galactose induction. **C** Lane profile analysis of blots in B. Y-axis represent the average of intensities of pixels with respect of the distance for each individual lane. Only results from the lanes with a MNase induction of 15 minutes are displayed. DNA cuts are indicated as in B. **D** Same as B, but strains with *sir4Δ* allele were tested.

All the ChEC analyses above concerned yKu associations with sites that contained telomeric repeats relatively close to an actual telomere, either terminal repeats or ITSs within about 10 kb of a telomere. If indeed telomeric repeats and associated proteins are replication fork barriers, they should cause replication blocks anywhere in the genome. In order to test this prediction, we inserted a plasmid containing either a 260 bp block of telomeric repeats (+ITS) or no such repeats (-ITS) into a non-telomeric area near the *HIS3* locus on chromosome XV, about 300 kb away from the telomere on XV-L (Fig 6A and S6A Fig). After performing ChEC with Yku70-MN and probing this locus, DNA cleavage at the predicted site was detected only in the +ITS situation (Fig 6B and S6B Fig). Furthermore, the cutting at this artificial ITS was specifically mediated by yKu, since when the ChEC was performed with GBD-MN, no cleavage near this constructed ITS was observed (Fig 6C). Finally, an ITS-dependent signal after Yku70-MN ChEC was also detected at this site in cells with a *sir4Δ* allele (Fig 6D). These data thus confirm the association of yKu with loci in which ITS occur and also show that a nearby telomere is not required for this association.

**Fig 7:**
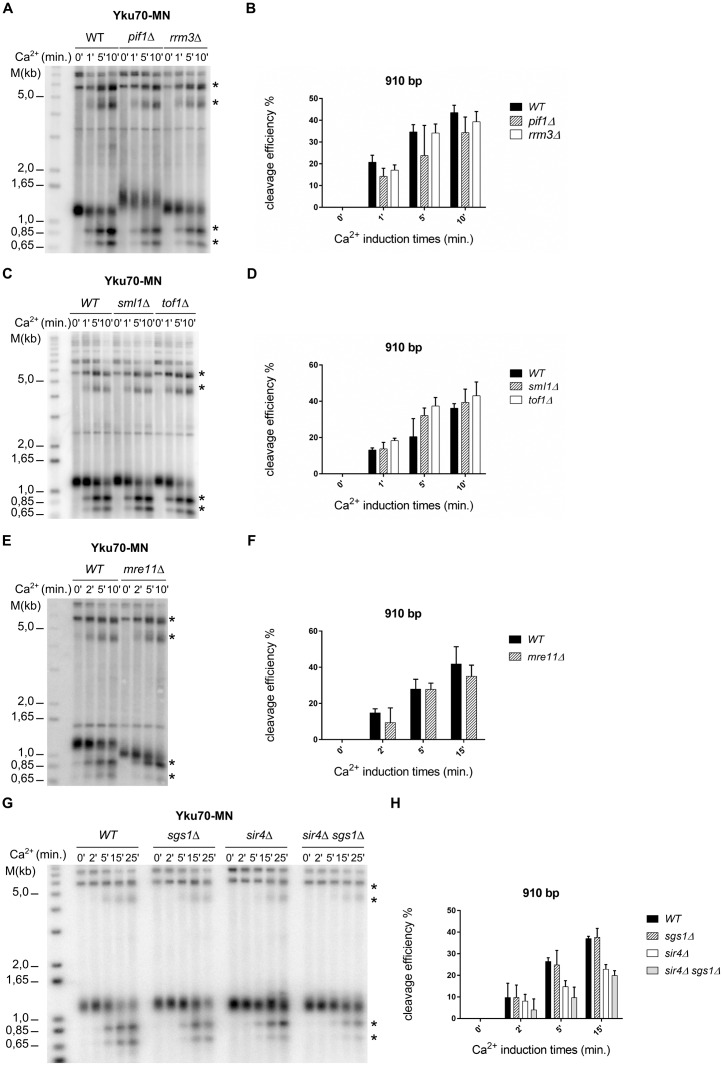
yKu70-MN mediated DNA cuts are independent of DNA replication associated helicases and replication stress sensors. **A** *In vivo* ChEC performed and analyzed in strains with indicated deletions for helicase genes (*pif1Δ* or *rrm3Δ*). Analysis of genomic DNA by XhoI digestion and hybridization to a Y’-specific probe was as in Fig 2. Symbol used for labeling of new fragments is *. **B** Quantification of the occurrence of the 910 bp fragment with respect of the time of Ca^2+^ addition. Differences between the different conditions as P values were calculated as in Fig 2C and indicate no statistical difference between the three samples at any time point. **C** and **D** same as A and B, but strains with the *sml1Δ* and *tof1Δ* alleles were tested. Quantification was realized on three independent experiments for *pif1Δ* and *rrm3Δ* mutants and on two independent experiments for *sml1Δ* and *tof1Δ* mutants. **E** and **F** same as A and B, but strains with the *mre11Δ* allele was tested. Quantification and statistics was as in Fig 2C and no difference was detected. **G** and **H** same as A and B, but strains harbouring *sgs1Δ*, *sir4Δ* or *sir4Δ sgs1Δ* alleles were tested. Quantification and statistics were as in Fig 2C. No statistical relevant differences were detected.

The Rrm3 and Pif1 helicases have been proposed to facilitate replication fork passage through telomeric repeat sequences and without them, fork stalling appeared more prevalent [29, 30]. However, Yku70-MN mediated cleavage near fork stalling sites was not increased in either *pif1Δ* or *rrm3Δ* strains (Fig 7A and 7B, S7A Fig). Strains with deletions of Tof1 or Sml1, although also predicted to be more susceptible to fork disassembly, displayed only marginally increased cleavage efficiencies as compared to wt, and these differences were not statistically significant (Fig 7C and 7D; S7B Fig). These results suggest that while actual fork stalling at ITS sequences may be sensitive to repeat orientation and replisome stability, the overall frequency of converting the stall to a one-sided break is not.

### yKu mediated DNA cleavage is not affected by an absence of the MRX complex but is slightly reduced in cells with an *sgs1Δ* mutation

Previously it was suggested that after yKu binding onto a DSB, 5’-strand resection mediated by the Mre11/Rad50/Xrs2 complex in preparation for homologous recombination may remove yKu from the DNA end [52]. If this was the case as well for the one-sided breaks that are expected to occur near replication arrests, in the absence of Mre11 we expected to observe an increase in Yku70-MN mediated cleavages near telomeric repeats. Hence, we constructed a strain harbouring an *mre11Δ* allele which did display short telomeres, as expected (Fig 7E, lane 0’). However, the Ca^2+^ dependent generation of the short telomeric fragments was not increased (Fig 7F and S7C Fig). If anything, there was a slight decrease in cleavage efficiency such that after 2 min with Ca^2+^, efficiencies for the two fragments were WT_910_: 14,7%; WT_770_: 7,1%; *mre11Δ*_*910*_: 9,3%; *mre11Δ*_*770*_: 4,2%. These results suggest that the Mre11/Rad50/Xrs2 complex does not play a role in yKu-release from the DNA sites near telomeric repeats analyzed here. Finally, strains harbouring a *sgs1Δ* allele or a combination of *sgs1Δ* with *sir4Δ* displayed slightly decreased cleavage efficiencies, albeit again not in a statistically significant manner (Fig 7G and 7H, S7D Fig).

## Discussion

The DNA binding complex Ku binds to dsDNA ends without any sequence specificity. This association occurs at a physical end of a DNA molecule and the DNA end will pass through a ring-like opening of Ku [3]. In the DNA-bound configuration, the majority of the Ku70 protein faces the side that is proximal to the DNA end, while the surface of the Ku80 protein faces towards the other side [3]. Previous data from budding yeast also suggested that this orientation had functional consequences: systematic screenings of mutations in both subunits showed that it is the Yku70 protein that is the major determinant for mediating NHEJ, which involves the physical DNA end side [20]. However, Ku also associates with telomeres in many organisms, including humans and yeast [2, 9]. At this location, NHEJ-induction could cause chromosome fusions with ensuing genome instability which must be avoided. Yet, how exactly NHEJ-induction by Ku is prevented at telomeres remains unknown.

In budding yeast, yKu is associated with telomeres in two ways: either the complex is bound directly to DNA, as on any DNA end and as described above, or it is associated indirectly via an interaction between Yku80 and the telomeric chromatin component Sir4 [17, 20, 47]. Previous results do show that yKu must be bound to the DNA directly in order to mediate NHEJ and the telomeric capping functions ascribed to it [16, 47]. Furthermore, yKu is associated with telomeres even in *sir4Δ* cells [40, 47] and the question of how NHEJ is prevented at that location remains.

Our data here show that yKu binding on telomeres can occur at sites that are distal from the physical ends of chromosomes, regardless of whether the cells contained Sir4 or not (Figs 2, 3 and 6). The sites that can be detected using the ChEC assay are near the telomeric repeat to subtelomeric DNA junctions and on ITSs (Figs 2 to 6). As expected from a general phenomenon not dependent on a specific genomic locus, yKu binding to ITSs was also detected on a chromosomal internal site (*HIS3* locus), far from a telomeric region (Fig 6). The fact that these internal associations are based on direct DNA binding is underscored by ChIP assays in which the signal is only completely lost if both, the ability of binding DNA (the *yku80Δ36* allele) and the interaction with Sir4 (in *sir4Δ* cells*)* are removed (Fig 2C). Consistent with this new placing of yKu on telomeres, the yKu complex can be detected on excised circular DNA that does not contain the most distal part of a modified telomere VIIL (Fig 4). This proposed localization of yKu is unexpected because it was assumed that yKu would bind to the telomeric DNA from the very ends of the chromosomes for its telomeric functions (see for example [16, 47]). We consider it highly unlikely that the detected internal binding reported here is due to end binding and then sliding of yKu on the DNA to its final position. This is particularly so for the binding detected on the ITSs, which would require yKu sliding on chromatinized DNA *in vivo* for at least 4 kb, or for about 300 kb in the case of the artificial ITS constructed at the *HIS3* locus (Figs 1 and 6). We therefore think it more plausible that yKu associates on DNA ends that were generated near the sites detected.

**Fig 8:**
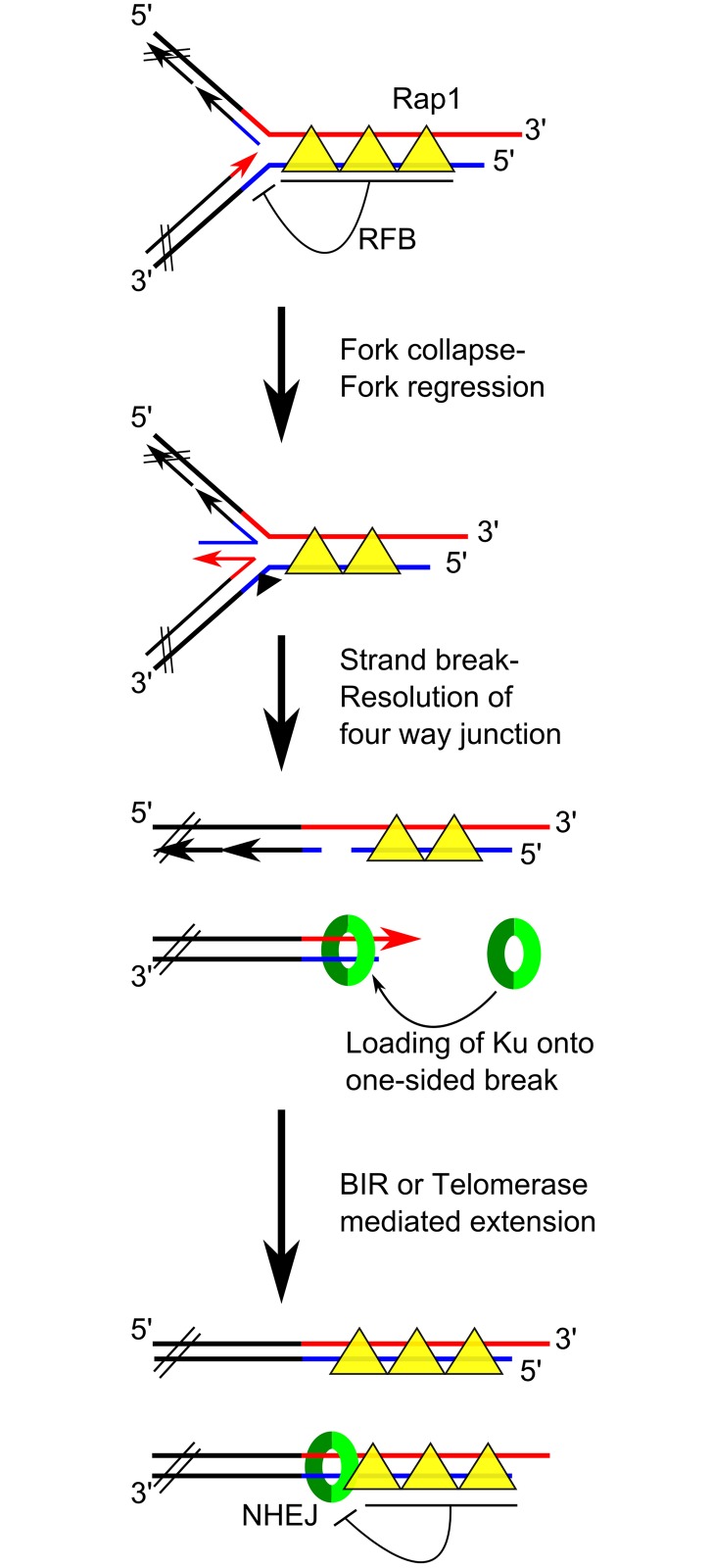
A model for yKu function at telomeres. The drawings depict a scenario that is consistent with all data and explains how the propensity of Ku to mediate NHEJ is averted at telomeres. DNA strands in black: subtelomeric sequences; DNA strands in red or blue: G-rich and C-rich telomeric repeat strands, respectively. Rap1 is the major telomeric repeat binding protein in budding yeast. RFB = Replication Fork Barrier.

The above raises the questions of how and why a DNA end is generated at ITSs and at the beginning of the terminal telomeric repeats. It is well documented that telomeric repeat sequences, including ITS tracts, can be major obstacles for the passage of a replication fork [27–30]. Furthermore, there is now direct evidence that stalled or stressed forks will generate a DNA double stranded break [31]. In line with this evidence, we propose that during S-phase, stalled replication forks near or in telomeric repeat tracts could reverse and/or be subject to strand breakages that would create what is dubbed a one-sided DSB (Fig 8). yKu could then bind those ends via its canonical binding mode on DNA. Depending on the precise end-structure generated by the break, the presence of yKu on them could prevent extensive resection but perhaps still mediate the initiation of break induced replication [53] or repeat extension by telomerase, which would secure the re-establishment of a functional telomere distal of the break. Our data also show that new yKu associations with ITSs requires that cells are growing and such new associations do not occur during G1 (Fig 4E and 4F, S4 Fig). Consistent with these results, *in vitro* binding studies showed that yKu cannot associate with a DNA end to which the telomeric capping protein Cdc13 was pre-associated [54]. Moreover, only when Cdc13 is actively degraded and removed from telomeres during G1 is there an end-stabilising effect exerted by yKu [55], as would be expected from the *in vitro* results [54]. However, yKu does not protect telomeric ends from degradation during late S-phase, when telomeric replication occurs *in vivo* [21, 55]. Formally, we cannot completely exclude the possibility that yKu associates with the non-terminal sites via an association that is dependent on as of yet unknown protein-protein interactions that do not involve Sir4. Arguing against this possibility is the finding that in *sir4Δ* cells that harbour the *yku80Δ36* allele [16], a *YKU80* allele that reconstitutes a yKu complex that is unable to bind to DNA, the yKu association with ITSs is completely lost (Fig 2C).

Remarkably, we can detect yKu at internal sites even if cells are in G1 of the cell cycle (Fig 4C and 4D). Given that new yKu associations with ITSs do not occur in G1 (Fig 4E and 4F), these results suggest that at least on the telomeric sites analyzed here, yKu removal is inefficient. Consistent with this idea, detection of yKu on a non-telomeric ITS appears much lower than that on telomeric ITSs (compare Figs 2B, 3A with 6B). We ignore the reason for these differences but suggest that different chromosomal locations may be differentially susceptible for yKu removal. This idea has precedence as phosphorylated H2A also appears to have a much longer persistence time in subtelomeric areas [56, 57]. In fact, the strong correlation of γ-H2A accumulation with replication barriers in telomeric areas [56] thus correlates with the accumulation of yKu on these same sites and reinforces our model (Fig 8). Recently it was proposed that yKu bound on DNA ends on a DSB would be removed by the nuclease activity of the MRX-Sae2 complex in preparation for homologous recombination (HR)[58]. Our data show that an absence of the Mre11 protein does not influence Yku70-MN mediated DNA cutting at telomeric sites (Fig 6). This finding correlates with the fact that HR is actively suppressed at telomeric loci [40, 59], a suppression that is lost in cells that lack yKu [10]. Furthermore, the nucleolytic activity of Mre11 is not required for its telomeric functions [60]. These considerations are consistent with our model that predicts that the nucleolytic activity of MRX-Sae2 for HR initiation is repressed on telomeric one sided breaks, essentially leaving yKu on the DNA. We do not know whether the MRX-complex still associates with these ends and how the potential ensuing BIR events are induced in this situation, but a recent study implied an MRX-tethering function may be important for this later step [61]. In our case, this MRX-mediated tethering as well as the possible recruitment of telomerase would be independent of the nuclease activity of Mre11. While not statistically significant, there is a trend for increased Yku70-MN cleavage in *tof1Δ* cells and a decrease of similar extent in *sgs1Δ* cells (Fig 7D, S7B Fig; Fig 7H and S7D Fig). These two genes had been found to be involved in replication barrier efficiency, albeit in different types of pathways [27]. The ChEC assay is relatively complex and not ideal for documenting smaller differences in cutting efficiencies. The significance of the above observations on *TOF1* and *SGS1* therefore remains unclear.

Our model hence posits an important function of yKu at replication barriers at the transition between non-telomeric and telomeric repeats DNA (Fig 8). Its binding could suppress extensive 5’-resection and mediate fork stability / fork restart by BIR, or the binding of Cdc13 and recruitment of telomerase for repeat expansion. It is important to note that yKu is not expected to mediate NHEJ in this situation: first, this would be a yKu association with a one-sided break and hence, another end for a fusion reaction is not readily available. Second, the regulatory networks directing DNA double-strand break repair choice strongly favour HR over NHEJ during late-S-phase [52]. We note that there is previous evidence for very similar non-canonical functions of Ku in fission yeast [62]. A Ku mediated stabilisation of one sided breaks occurring near telomeres also impinges on a very important conundrum in the field, namely that of accommodating Ku-binding at telomeres and at the same time complete repression of NHEJ involving chromosome ends. If yKu is trapped on DNA relatively distant from the physical end, the binding of Rap1 proteins between yKu and the actual end could prevent yKu mediated NHEJ at telomeres. This in turn would also explain why Rap1 has a strong NHEJ repressing effect [63]. However, our results do not directly address the issue of whether or not there is yKu binding at the physical ends of chromosomes, where the Cdc13/Stn1/Ten1 complex in conjunction with a specialised Rap1-based chromatin provide for the essential capping function.

## Methods

### Strains

Full genotypes of all strains are described in S1 Table. We constructed yeast strains EPY007 and MVY221 expressing MN-Rap1 and Yku70-MN respectively by fusing the enzymatic activity domain of micrococcal nuclease (MN) from *Staphylococcus aureus* to the N-terminus of the Rap1 protein or the C-terminus of Yku70. NruI linearized plasmid pRS306-MN-Rap1 was transformed into a diploid wt strain (W303). Cells that had lost the *URA3* marker were then selected by restreaking one isolated colony on an FOA plate. The resulting diploid strain was sporulated and clones expressing the fusion protein were identified. Strain Yku70-MN was obtained with PCR based mutagenesis using primers flanking by the C-terminal sequences of *YKU70*, F2-Hdf1 and R1-Hdf1 with plasmid pFA6a-MN-TRP1 as template. The fragment was used for transformation of diploid strain MVY60 which subsequently was sporulated and clones expressing the Yku70-MN allele were identified. All strain constructs were verified by southern blotting. For expression of GBD-MN, strain W3749-1a was transformed with the replicative pRSE plasmid that contains the *TRP1* marker and the Gal4 DNA binding domain fused to Micrococcal nuclease ORF, the gene being transcribed from the yeast *GAL1* promoter. Strain MVL013 was derived from MVY221 in which the *BAR1* gene was replaced by a *natMX4* by PCR-mediated gene disruption [64, 65].

Strains MVL022 and MVL023 were derived from MVL013 in two steps. First we integrated a construct such that the 2μ-Flip protein could be induced by galactose using plasmid pFV17 [66]. This strain was then transformed with the linearized plasmids sp225 and sp229 to obtain respectively strains MVL022 and MVL023, as described previously [49]. Strains MVL047 and MVL048 were derived from MVL013 by replacing the *SIR4* gene by the *kanMX4* [67]. Strain MVL052 was derived from MVL013 by replacing the wt *YKU80* gene by the *kanMX4*. Strain MVL052 was then transformed with either the pML7c-2 plasmid, which contains the wild type *YKU80* locus including its endogenous promoter, or the pML7c-14 plasmid, which contains the *yku80L140A* allele with its native promoter. Strain MVL010 was derived from MVY221 by replacing the *SIR2* gene with the *natMX4* deletion cassette [64]. Strain MVL054 was derived from EPY007 by replacing the *SIR4* gene with the kanMX4 deletion cassette. For analysis of linear plasmids, BamHI-linearized plasmids YLpRW40-2 and YLpRW41 [51] were transformed into MVL013 or into W3749-1a + pRSE. Clones were selected on Yc-URA-LEU plates or on Yc-URA-LEU-TRP plates. Strains MVL030, MVL031, MVL032 and MVL033 were derived from MVL023 by replacing, respectively, the *PIF1*, *RRM3*, *SML1* and *TOF1* genes with the *kanMX4* deletion cassette. Strain MVL063 was derived from MVL013 by replacing the *MRE11* gene with a *HIS3* auxotrophic marker [68]. Strains EPY050 and EPY052 were derived from, respectively, MVL013 and MVL047 by replacing the *SGS1* gene with a *URA3* deletion cassette. Strains EPY027, EPY028 and EPY031 were derived, respectively, from MVL023, MVL022 and W303, by replacing the *SIR4* gene with the k*anMX4* deletion cassette. All modifications in the genome of the above mentioned strains were verified by colony PCR and Southern blotting using a probe that hybridizes to the promoter of the respective gene. Strains EPY054, EPY058, EPY061 and EPY064 were obtained from, respectively, strains W303, EPY031, MVL013 and MVL047, transformed with NheI linearized plasmid pRS303. Strains EPY056, EPY059, EPY063 and EPY066 were obtained from, respectively, strains W303, EPY031, MVL013 and MVL047, transformed with NheI linearized plasmid pEP19A. Strains EPY054, EPY058, EPY056 and EPY059 were then transformed with pRSE plasmid.

IDY80-1 strain was derived from MLY30 in which the *YKU80* gene was replaced by the *LEU2* gene. The *SIR4* gene was replaced in this strain by *natMX4* cassette, to obtain IDY82-9 strain. Strains IDY80-1 and IDY82-9 were transformed with either pJP7c (YKU80-myc) or pJP12 (yku80Δ36-myc) to perform ChIP experiments. Inducible myc tagging of Yku80 experiment was done in strain IDY80-1 transformed with YCpHOCut4 and either pEP22B or pEP24C.

### Plasmids

All plasmids are described in S3 Table. pRS306-MN-Rap1 was derived from pRS306 [68] by insertion of three fragments; i) the gene-proximal last 490 bp of the Rap1 promoter (Rap1 promo), ii) a DNA fragment encoding the enzymatic domain of micrococcal nuclease, iii) the first 500 bp of the *RAP1* coding region (Rap1-500). The Rap1 promo and Rap1-500 fragments were amplified by PCR from genomic DNA with the following primers; Rap1 Promo XhoI For/ Rap1 promo ClaI Rev and Rap1 500 bp ClaI For/ Rap1 500 bp EcorI Rev respectively (see S2 Table for details on all primers). First, both of these fragments were integrated simultaneously into the EcoRI-XhoI sites of pRS306. A second cloning step permitted to integrate the MN-encoding fragment into the ClaI restriction site. This latter fragment was amplified by PCR from pFA6a-MN-TRP1 [36]. pRSE was derived from the pRS314-Cre-EBD plasmid. First, pRS314-Cre-EBD was obtained by inserting the Gal1-Cre-EBD fragment from pSH62-EBD [69] into the SacI-EcoRI sites of pRS314. The Cre-EBD fragment was removed from pRS314-Cre-EBD by EcoRI-SalI digestion. The fragments encoding the Gal4 DNA binding domain (GBD) fragment and the MN were inserted into this plasmid by Gibson Assembly [70]. pML7c-2 and pML7c-14 plasmids were derived from pJP7c and pJP7c-L140A plasmids respectively. pJP7c is derived from the pJP7 plasmid [16] in which a point mutation had to be corrected. Essentially, these two plasmids comprise the pRS313 backbone into which either the wild type *YKU80* locus or the *yku80L140A* allele with its native promoter were inserted. Both proteins were tagged with two Myc and ten HIS tags. YRpRW40-2 was derived from YRpRW40 by correcting the internal telomeric repeat tract to be the same as in YRpRW41. pEP19A was derived from pRS303. A 256 bp telomeric track was integrated between the XbaI and BamHI sites. pEP22B and pEP24C were derived from pJP7c and pJP7c-L140A respectively by insertion of the bacterial *recR* gene transcribed from the yeast *GAL1* promoter. The *YKU80* alleles also contained two RS sites upstream of the Myc and HIS tags (see S4 Fig). Nucleotide sequences of these plasmids are available upon request.

### Yeast growth

All culture growth was at 30°C in standard yeast cell growth conditions (YEP media with indicated carbon sources or in drop-out media). Strains with pRSE plasmid were pre-grown in Yc-TRP media with 2% raffinose to stationary phase. Cells were then diluted and grown in Yc-TRP with 2% galactose for 45 minutes up to 3 hours. Strains MVL022 and MVL023 were pre-grown in YEP media with 2% raffinose to stationary phase, the culture it was diluted in YEP media with 2% raffinose and re-grown to an OD_660_ of ~ 0.4. A first asynchronous/FLP non-induced aliquot was left to grow to an OD_660_ of ~ 0,6. A second aliquot was grown with 2% galactose to an OD_660_ of ~ 0,6 (asynchronous/FLP induced sample). To a third fraction, we first added α-factor (final 0.1 μM) for 90 mins. The culture was verified for G1 arrest by FACS analysis. After this treatment, one aliquot was grown with 2% galactose to an OD_660_ of 0,6 (synchronous/ FLP induced sample). The rest of the culture was left to grow to an OD_660_ of ~ 0,6 in the presence of glucose (synchronous/FLP non-induced sample). The ChEC assay was then performed on all samples.

### *In vivo* ChEC: Chromatin endogenous cleavage using living cells

From an overnight pre-culture, cells were diluted into 100 ml media and re-grown to an OD_660_ of about 0.6–0.8. Cells were harvested and washed three times in 1 ml A-PBPi buffer [36]. Cells were permeabilized in 600μl Ag-PBPi buffer for 5 min at 30°C. For MN-cleavage, CaCl_2_ was added to a final concentration of 2 mM and cells incubated at 30°C. A first aliquot was taken before Ca^2+^ addition for time point 0, and the next aliquots were removed at indicated time points after the addition of Ca^2+^. Aliquots were immediately mixed with an equal volume of a 2X STOP solution (400 mM NaCl; 20 mM EDTA; 4 mM EGTA; 0.2 μg/μl glycogen).

### DNA isolation, southern-blotting and in-gel analyses

Cells were mechanically broken using glass beads and DNA extraction was realized as described previously [36]. Appropriate quantities of DNA were digested with indicated restriction enzymes, separated on 0.6% TBE agarose gels, transferred on a Hybond-XL nylon membrane (Amersham) and detected by hybridization with 32P-labelled radioactive probes. 500 ng of digested DNA was loaded on gels for hybridization with Y’-specific probe and telomeric repeats probe, and up to 2.5 μg for hybridization with other specific probes. Blots were analysed using the Typhoon FLA 9500 from GE Healthcare Life Sciences. Band intensities for cleavage efficiencies were quantified with Image quant software. For each fragment, the cleavage efficiency percentage is calculated with respect to total signal at each time. Cleavage efficiency = (fragment signal (tX)/ total signal) *100. Native in-gel analysis was performed as described [71]. As controls, DNAs derived from a wild type or a strain with a *yku70Δ* allele were used. After hybridization and washings, the gel was exposed to MP-high performance film (Amersham) for appropriate times. For loading controls, the DNA was then transferred to Nylon membranes which were hybridized to a probe with telomeric repeats.

### Chromatin immunoprecipitation

Chromatin immunoprecipitation (ChIP) experiments were performed essentially as described [72] with some modifications. Briefly, cells were grown to an OD_600_ of 0.5–0.6. Formaldehyde solution (37%) was added to a final concentration of 1% and cells incubated for 20 min at room temperature. Cell pellets from 50 ml cultures were resuspended in 500 ml of lysis buffer containing proteases inhibitors and disrupted vigorously with glass beads three times for 30s using a FastPrep-24 (MP Biomedicals) instrument. Samples were then sonicated 10 times for 10s at 20% power using a Branson digital sonifier. Whole-cell extracts were incubated with anti-myc (9E10, Roche) antibody overnight at 4°C, and precipitated with Pro-A/G Magnetic Beads (Pierce) for 1 hour at 4°C. Quantification of the immunoprecipitated DNA was accomplished by quantitative real-time PCR, employing the SYBR Green (Life Technologies) system. Immunoprecipitated DNA was normalized to input samples to calculate the percentage of input DNA that was precipitated. Control qPCR assays were targeted to the *CLN2* locus to demonstrate non-amplification of non-target loci.

For the arrested and galactose induced analyses, cells were pre-grown in raffinose and arrested with 0.1 μM α-factor (Sigma), arresting for 4 hours. Half the culture was washed and released into media containing Pronase (Roche) and galactose. To the remaining half of the culture, galactose was added. Galactose induction proceeded for 10 hours before cells were harvested for ChIP as described above.

### Protein extracts and western blotting

Whole cell protein extracts were prepared as previously described [73]. Samples were analyzed on 8% SDS-PAGE followed by electroblotting onto HybondECL membrane (GE-Healthcare). Membranes blocked in 5% milk/PBS-T were incubated in 1:1000 monoclonal rabbit anti-Myc antibody (Cell signalling) diluted in 1% milk/PBS. Secondary antibodies were donkey anti-rabbit (GE Healthcare), diluted 1:5000 in 1% milk/PBS. Blots were visualized and analyzed on a LAS-4000 (GE Healthcare).

## Supporting Information

S1 Fig General principle and example for the Chromatin Endogenous Cleavage (ChEC) method. **A** Schematic of the method in which a DNA-binding protein of interest is fused with Micrococcal Nuclease (MN). Upon addition of 2 mM Ca^2+^ to living cells, the MN becomes active and induces DNA double strand breaks in the vicinity of the DNA site of the protein. These Ca^2+^ dependent cuts can be visualized by Southern blotting and probing of adequately digested total cellular DNA. **B** Complete digestion control for the blot shown in Fig 1A. The blot was re-hybridized with a probe specific for 2 μm DNA that should detect a 3.2 kb fragment (indicated by an asterix). **C** Example of ChEC with MN-Rap1 on a genomic locus on chromosome II with two known Rap1-binding sites. Without Ca^2+^ (lane 0'), the genomic fragment remains intact at 6.6 kb. After Ca^2+^ addition, fragments of 6.1 kb and 4.3 kb, marking the Rap1-binding sites can be detected (see schema to the right).
(PDF)

S2 Fig Quantification of Ca^2+^ induced cleavage sites. **A-C** Graphs depict the occurrence of cleavage that generated the 770 bp fragments on the Southern blots shown in Fig 3. Differences between the different conditions as P values were calculated as in Fig 2. P values were calculated for three independent experiments as Fig 3.
(PDF)

S3 Fig ChEC with MN-Rap1 on a strain with a *sir4*Δ allele. **A** Same ChEC analysis as in Fig 3A, but the strain harboured the MN-Rap1 fusion protein. **B** Differences between the different conditions as P values were calculated as in Fig 2. P values were calculated for three independent experiments as Fig 3. **C** Same experiment as in Fig 4B and 4D, comparing a *SIR4wt* strain to one that harbours a *sir4Δ* allele. All cultures were non-synchronized and induced for episome release (grown to log-phase in galactose media). "ChrVIIL—0": no telomeric repeats on excised episome; "ChrVIIL—1": 270 bp of repeats on the episome. Note that in the *SIR4* strain, gal-induced flip-out was partial, explaining the two bands around 1.5 kb. Labeling of the gel as in Fig 4B and 4D.
(PDF)

S4 Fig An inducible Yku80 tagging system for assessing yKu binding on ITS in a cell cycle dependent manner. **A** pEP22B contains the *YKU80* gene with its native promoter as well as the RecR recombinase gene expressed from a GAL promoter. Before galactose induction (growth in glucose or raffinose), wildtype untagged Yku80 fused to a small peptide encoded by one “RS” sequence is expressed (left drawing). After galactose induction, the recombinase is expressed, the RS sequences undergo recombination resulting in a fusion of the myc-his tags to the Yku80 sequence (right). **B** A southern blot of DNA digested by NcoI and HindIII, demonstrating galactose inducible site-specific recombination between the RS sites at the end of the *YKU80* gene. Glu; Raf: cells were grown with glucose or raffinose as carbon source and only native pEP22B without RS pop-out is observed. Gal: addition of galactose and growth for the indicated hours. After 16 hours, the majority of pEP22B has undergone recombination. Probe used is indicated by a solid black line in A. **C** A western blot of whole cell protein extracts from the same cells as in B, demonstrating gal-dependent Yku80-myc expression from pEP22B. pJP7c expresses Yku80-myc constitutively and is used as positive control. Note the detection of Yku80-myc after 16 hours. Western blot was probed with an anti-myc antibody. **D** A graphical depiction of the *in vivo* situation used in this assay. *YKU80* is replaced in the genome with a *LEU2* marker. Oligos used for qPCR are shown as arrows on sites where they localize on the telomeres of ChrXII as well as YCpHOCut4. Cells contain both pEP22B (or pEP24C with the yku80-L140A version) and YCpHOCut4 (GAL-HO and Ho cut site). **E** Workflow for Arrest and Induction before ChIP analysis. Cells were grown to OD 0.5 in Raffinose. α-factor was added to a final concentration of 0.1 μM. Cells were allowed to arrest for 4 hours. An “Untagged” sample was taken after completion of the arrest. The remaining culture was split in half. Half was washed and released into media containing pronase and 2% galactose. Galactose was added to the other half to a final concentration of 2%. Galactose induction was allowed to proceed for 10 hours before all samples were harvested for ChIP analysis. **F** Experiment was carried out as in E with a strain containing pEP24C (yku80-L140A-RS-RS-myc) and YCpHOCut4. yKu localization to the HO cut-site on YCpHOCut4, as assessed by ChIP, was determined concurrently with assessment of localization to ITSs (Fig 4E) and the CLN2 locus. All IP samples were normalized to their respective input.
(PDF)

S5 Fig Details of the YLpRW40-2 and YLpRW41 plasmids and quantification of the cleavages occuring on them after ChEC with Yku70-MN. **A** Structure of the circular plasmids (left) which can be digested with BamHI to linearize them (right) in a way such that both ends contain an appropriately oriented telomeric repeat tract. Upon transformation of yeast cells, these ends will be used to form functional telomeres in vivo thereby establishing linear plasmids. The internal telomeric repeat tract is indicated as a black box and the analysed StuI-XhoI fragment is highlighted (see also Fig 5A). Note the localization of the ARS element on opposite sides of the analysed internal telomeric repeat containing fragment. As a consequences, the replication forks move in opposite direction through that internal fragment in the two plasmids. **B** Quantification of the occurrence of cleavages generating a 1060 bp and a 1160 bp fragment on the Southern blotting shown in Fig 5B. Differences between the different conditions as P values were calculated as in Fig 2. P values were calculated for three independent experiments as Fig 3.
(PDF)

S6 Fig Confirmation of specific cleavage by Yku70-MN on ITS far from chromosomal ends. **A** Schematic drawing of the modified *HIS3* locus that is 350 kb from the telomere on the right arm of chromosome XV (as in Fig 6A but with the localization of the NcoI/AatII restriction sites). Probe used in B is indicated by a solid black line. **B** In vivo ChEC with Yku70-MN on the left and GBD-MN on the right analyzed on locus depicted in A. Same genomic DNA that used in Fig 6B was digested with NcoI and AatII. The Southern blot was hybridized with a *MRM1* specific probe. Time of MNase induction by the addition of Ca^2+^ is indicated on top of the gel. Ca^2+^ induced cutting is indicated by a * for Yku70-MN. **C** Lane profile analysis of the 15 min lanes in B and as in Fig 6C. **D** Schematic drawing of the modified *HIS3* locus (as in Fig 6A) analysed in E. **E** Genomic DNA from an independent ChEC experiment was digested with NsiI. The Southern blot was hybridized with a HIS3 probe, depicted in D as a solid black line. Time of MNase induction by the addition of Ca^2+^ is indicated on top of the gel. Ca^2+^ induced cutting is indicated by a * for Yku70-MN. F Cleavage efficiency by Yku70-MN on ITS with two different probes (MRM1 (B) and HIS3 (E). Quantification was done as described in materials and methods.
(PDF)

S7 Fig Additional quantification of Ca^2+^ induced cleavage sites in mutant strains containing *pif1*Δ, *rrm3*Δ, *sml1*Δ, *tof1*Δ, *mre11*Δ or *sgs1*Δ alleles. **A-D** Graphs depict the occurrence of cleavage that generated the 770 bp fragment on the Southern blots shown in Fig 7. Significance differences between the WT and mutants strains (P values) were calculated as in Fig 2. Three independent biological replicas were performed for *pif1Δ*, *rrm3Δ*, *mre11Δ* and *sgs1Δ* alleles and two for *sml1Δ* and *tof1Δ*, as Fig 7. P values for the data in A and B indicate no significant differences.
(PDF)

S1 Table Yeast Strains Used. (DOCX)

S2 Table Oligonucleotides Used. (DOCX)

S3 Table Plasmids Used. (DOCX)
