# Supplementary figures and images for: Ku Binding on Telomeres Occurs at Sites Distal from the Physical Chromosome Ends
Source: PLoS Genet. 2016 Dec 8;12(12):e1006479. doi: 10.1371/journal.pgen.1006479 (PMC5145143; doi:10.1371/journal.pgen.1006479)

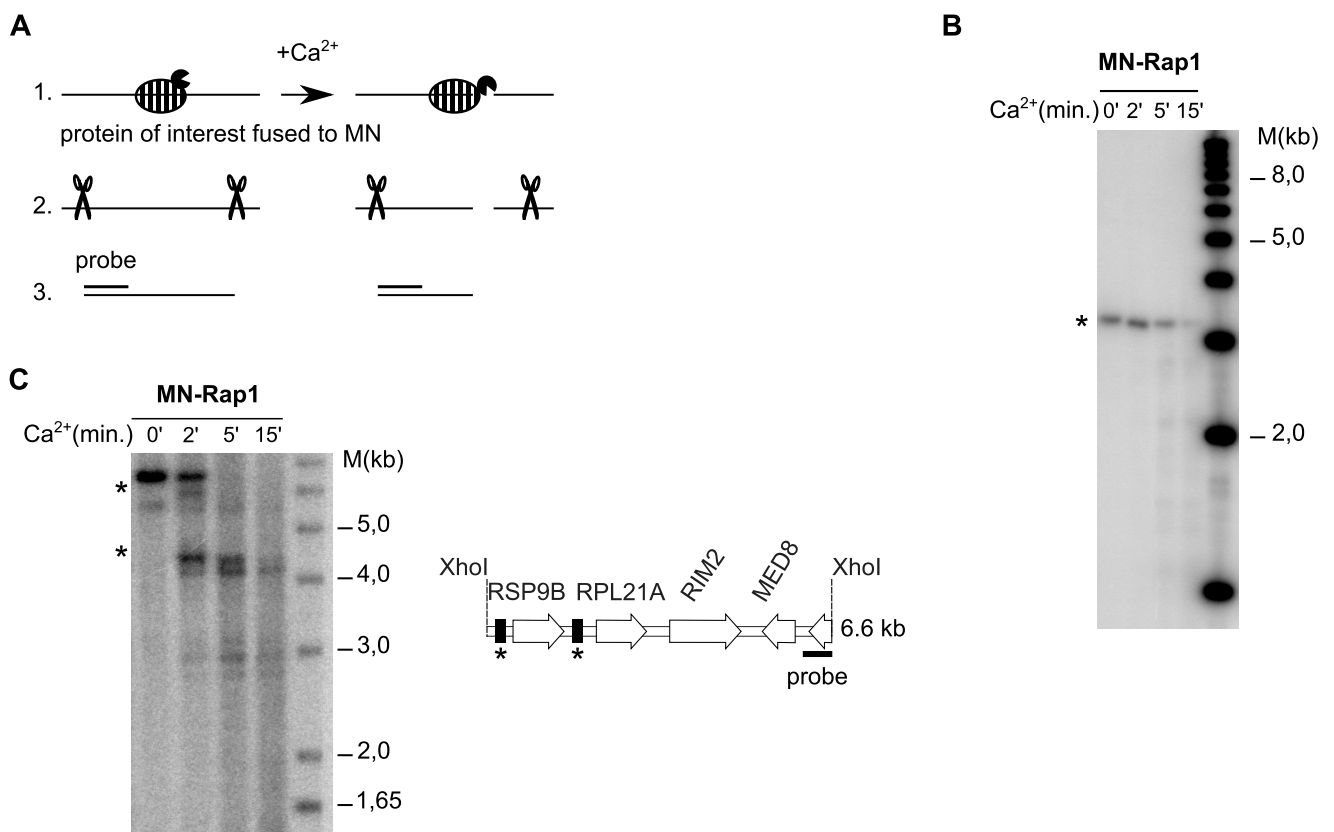

S1 Figure ; Larcher *et al.* 2016

Supplement: S1 Fig — A Schematic of the method in which a DNA-binding protein of interest is fused with Micrococcal Nuclease (MN). Upon addition of 2 mM Ca2+ to living cells, the MN becomes active and induces DNA double strand breaks in the vicinity of the DNA site of the protein. These Ca2+ dependent cuts can be visualized by Southern blotting and probing of adequately digested total cellular DNA. B Complete digestion control for the blot shown in Fig 1A. The blot was re-hybridized with a probe specific for 2 μm DNA that should detect a 3.2 kb fragment (indicated by an asterix). C Example of ChEC with MN-Rap1 on a genomic locus on chromosome II with two known Rap1-binding sites. Without Ca2+ (lane 0'), the genomic fragment remains intact at 6.6 kb. After Ca2+ addition, fragments of 6.1 kb and 4.3 kb, marking the Rap1-binding sites can be detected (see schema to the right). (PDF) [file pgen.1006479.s001.pdf]

**A**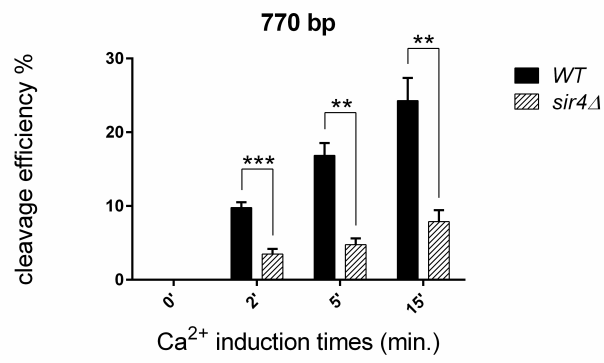**B**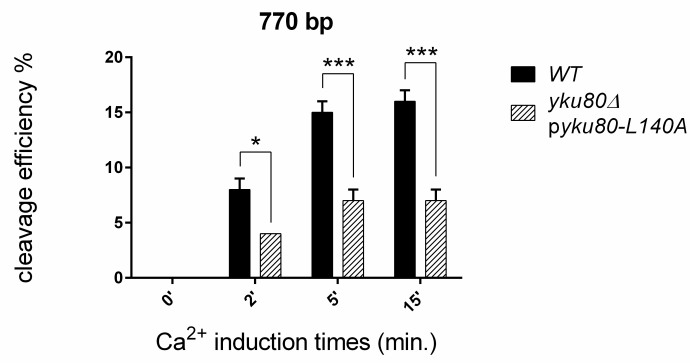**C**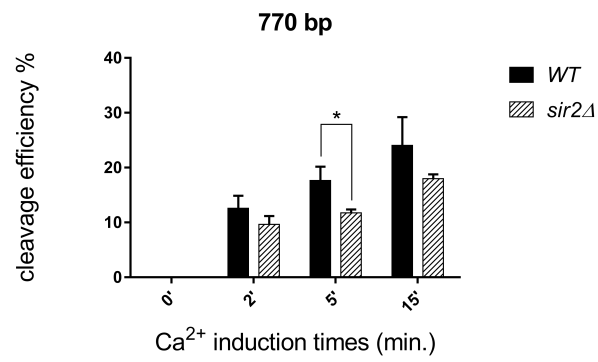S2 Figure ; Larcher *et al.* 2016

Supplement: S2 Fig — A-C Graphs depict the occurrence of cleavage that generated the 770 bp fragments on the Southern blots shown in Fig 3. Differences between the different conditions as P values were calculated as in Fig 2. P values were calculated for three independent experiments as Fig 3. (PDF) [file pgen.1006479.s002.pdf]

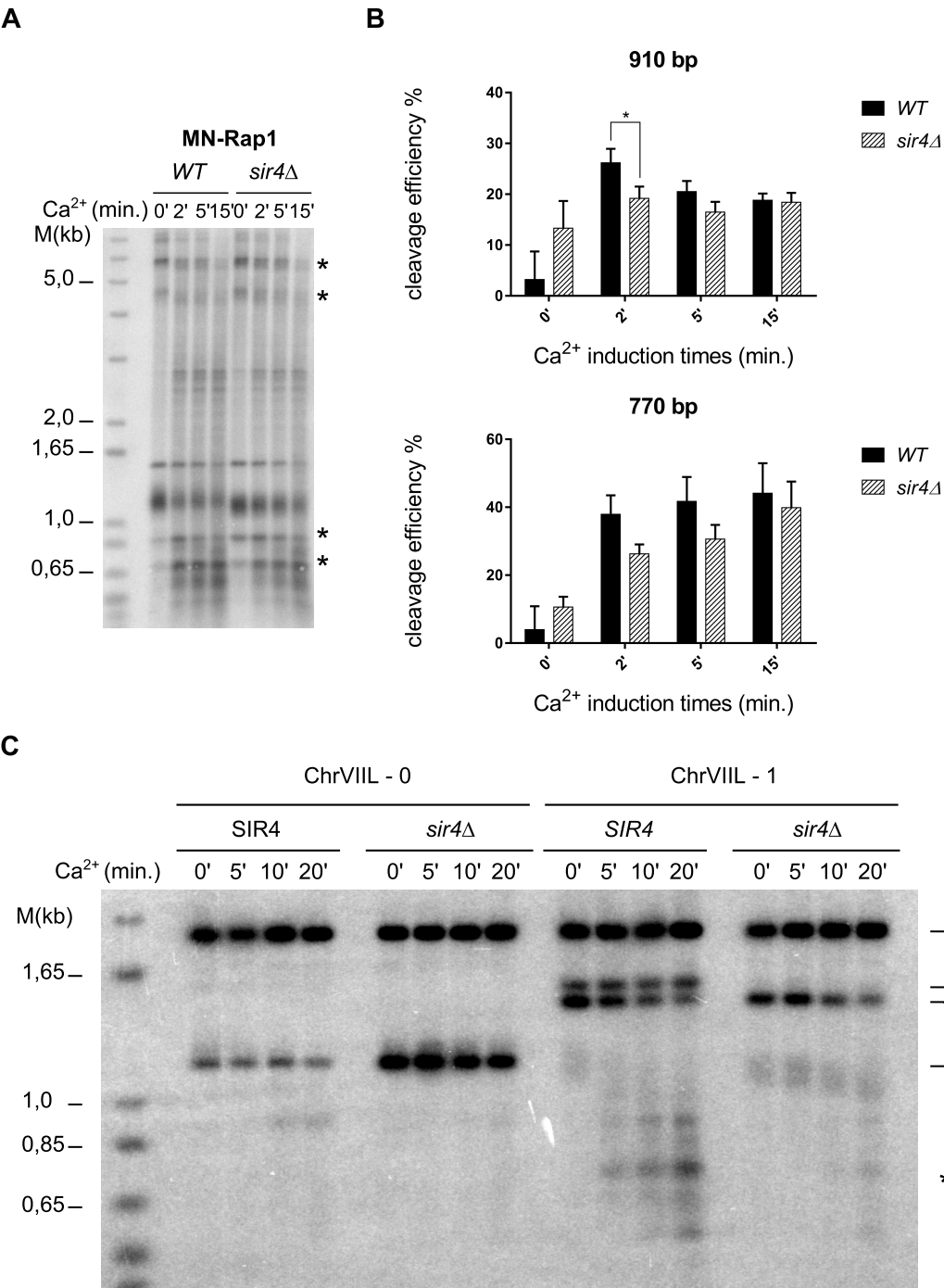

S3 Figure ; Larcher *et al.* 2016

Supplement: S3 Fig — A Same ChEC analysis as in Fig 3A, but the strain harboured the MN-Rap1 fusion protein. B Differences between the different conditions as P values were calculated as in Fig 2. P values were calculated for three independent experiments as Fig 3. C Same experiment as in Fig 4B and 4D, comparing a SIR4wt strain to one that harbours a sir4Δ allele. All cultures were non-synchronized and induced for episome release (grown to log-phase in galactose media). "ChrVIIL—0": no telomeric repeats on excised episome; "ChrVIIL—1": 270 bp of repeats on the episome. Note that in the SIR4 strain, gal-induced flip-out was partial, explaining the two bands around 1.5 kb. Labeling of the gel as in Fig 4B and 4D. (PDF) [file pgen.1006479.s003.pdf]

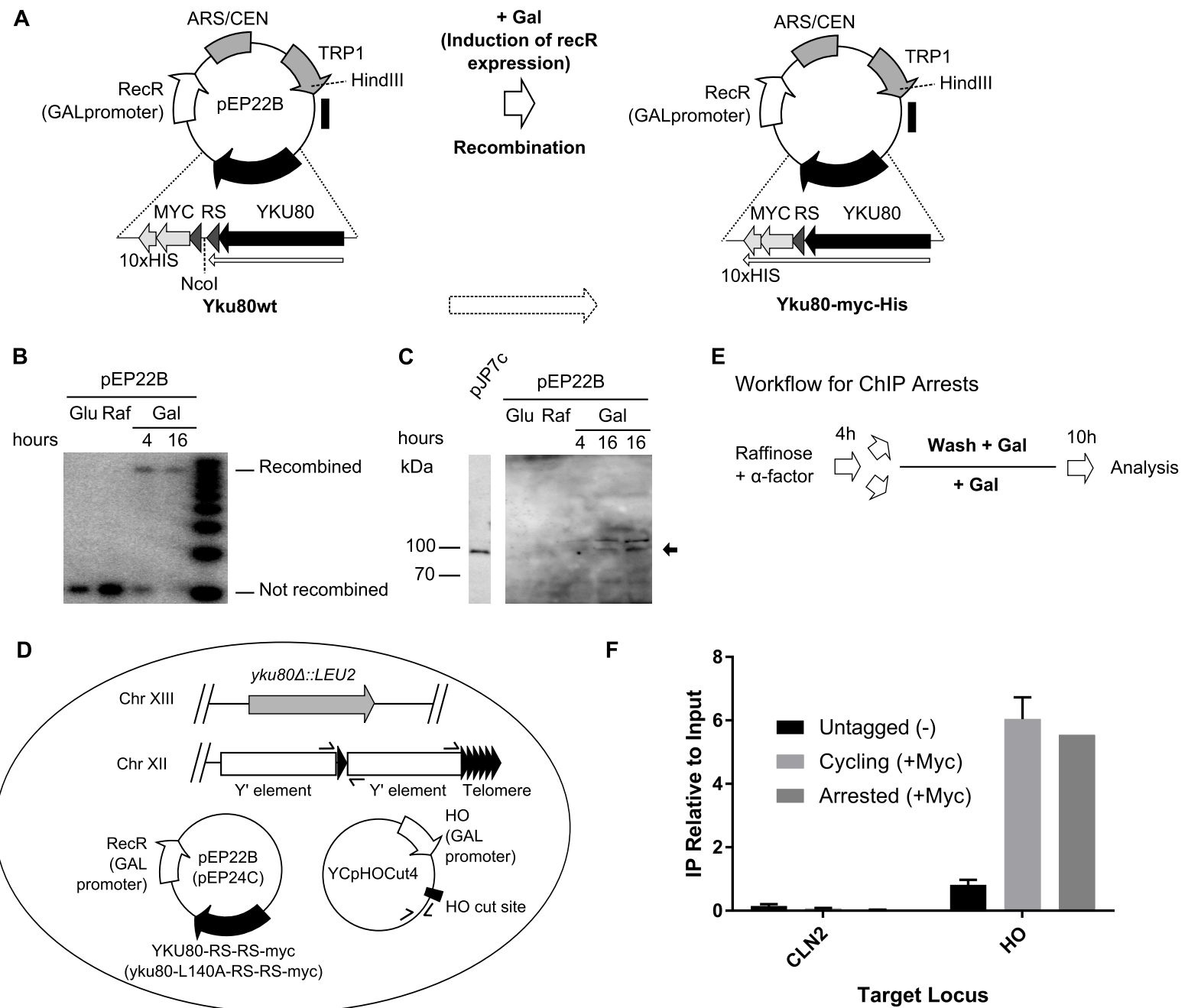

S4 Figure ; Larcher *et al.* 2016

Supplement: S4 Fig — A pEP22B contains the YKU80 gene with its native promoter as well as the RecR recombinase gene expressed from a GAL promoter. Before galactose induction (growth in glucose or raffinose), wildtype untagged Yku80 fused to a small peptide encoded by one “RS” sequence is expressed (left drawing). After galactose induction, the recombinase is expressed, the RS sequences undergo recombination resulting in a fusion of the myc-his tags to the Yku80 sequence (right). B A southern blot of DNA digested by NcoI and HindIII, demonstrating galactose inducible site-specific recombination between the RS sites at the end of the YKU80 gene. Glu; Raf: cells were grown with glucose or raffinose as carbon source and only native pEP22B without RS pop-out is observed. Gal: addition of galactose and growth for the indicated hours. After 16 hours, the majority of pEP22B has undergone recombination. Probe used is indicated by a solid black line in A. C A western blot of whole cell protein extracts from the same cells as in B, demonstrating gal-dependent Yku80-myc expression from pEP22B. pJP7c expresses Yku80-myc constitutively and is used as positive control. Note the detection of Yku80-myc after 16 hours. Western blot was probed with an anti-myc antibody. D A graphical depiction of the in vivo situation used in this assay. YKU80 is replaced in the genome with a LEU2 marker. Oligos used for qPCR are shown as arrows on sites where they localize on the telomeres of ChrXII as well as YCpHOCut4. Cells contain both pEP22B (or pEP24C with the yku80-L140A version) and YCpHOCut4 (GAL-HO and Ho cut site). E Workflow for Arrest and Induction before ChIP analysis. Cells were grown to OD 0.5 in Raffinose. α-factor was added to a final concentration of 0.1 μM. Cells were allowed to arrest for 4 hours. An “Untagged” sample was taken after completion of the arrest. The remaining culture was split in half. Half was washed and released into media containing pronase and 2% galactose. Galactose was added to th [file pgen.1006479.s004.pdf]

**A**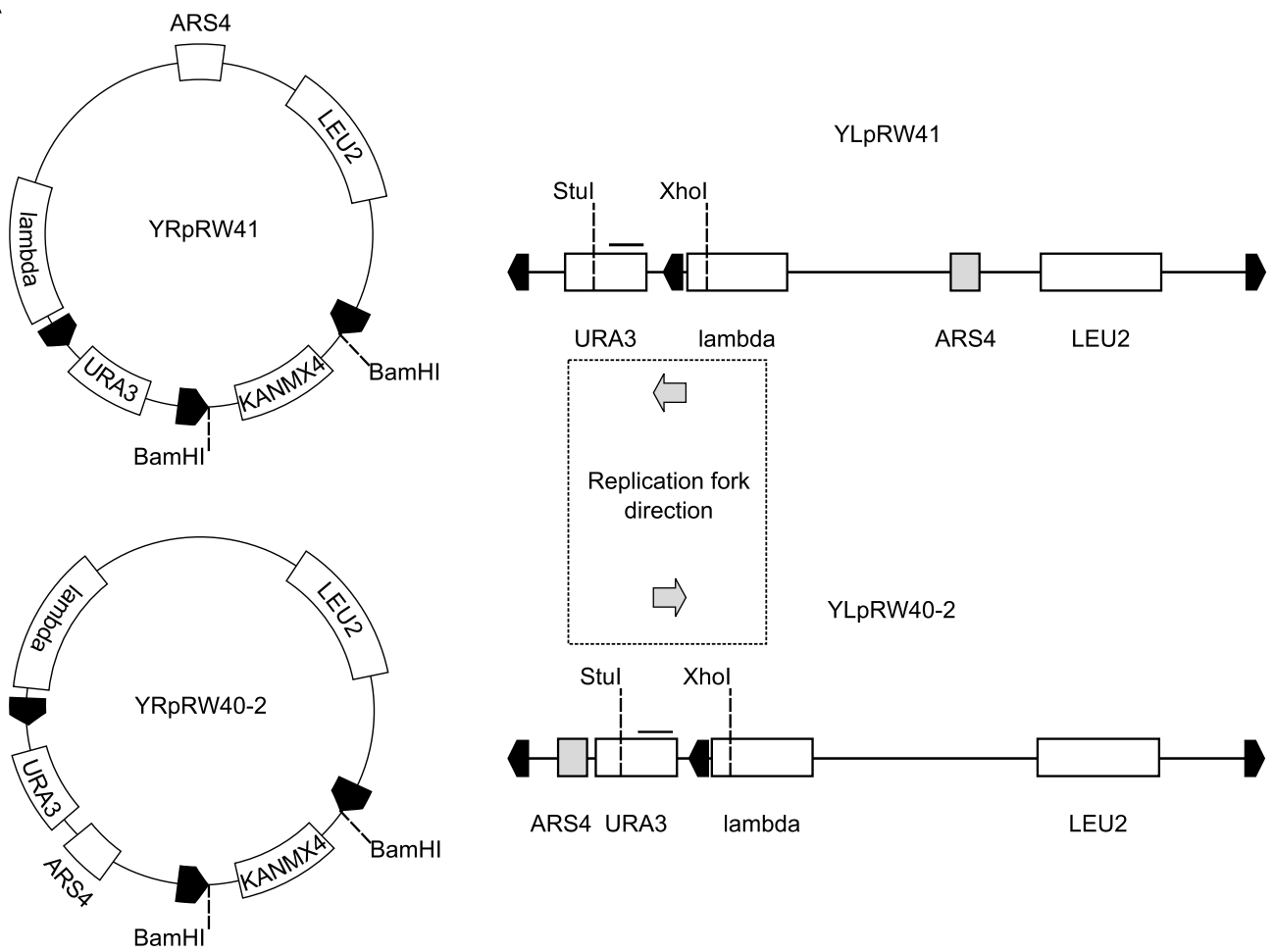**B**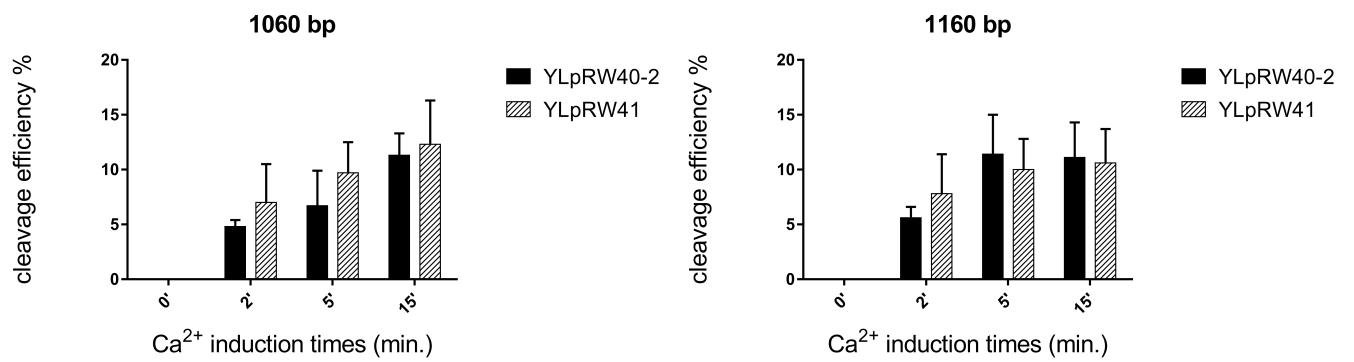S5 Figure ; Larcher *et al.* 2016

Supplement: S5 Fig — A Structure of the circular plasmids (left) which can be digested with BamHI to linearize them (right) in a way such that both ends contain an appropriately oriented telomeric repeat tract. Upon transformation of yeast cells, these ends will be used to form functional telomeres in vivo thereby establishing linear plasmids. The internal telomeric repeat tract is indicated as a black box and the analysed StuI-XhoI fragment is highlighted (see also Fig 5A). Note the localization of the ARS element on opposite sides of the analysed internal telomeric repeat containing fragment. As a consequences, the replication forks move in opposite direction through that internal fragment in the two plasmids. B Quantification of the occurrence of cleavages generating a 1060 bp and a 1160 bp fragment on the Southern blotting shown in Fig 5B. Differences between the different conditions as P values were calculated as in Fig 2. P values were calculated for three independent experiments as Fig 3. (PDF) [file pgen.1006479.s005.pdf]

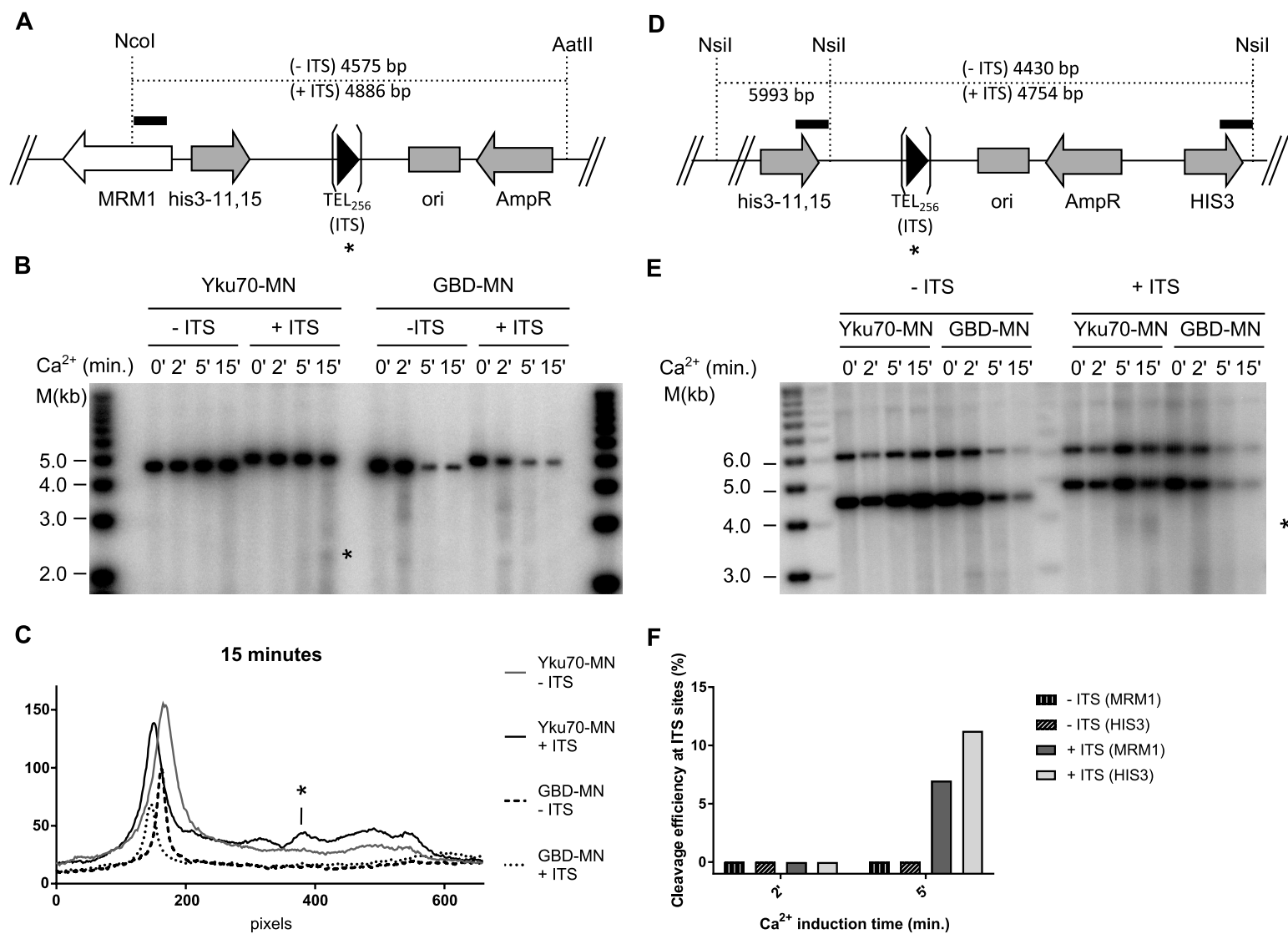

S6 Figure ; Larcher *et al.* 2016

Supplement: S6 Fig — A Schematic drawing of the modified HIS3 locus that is 350 kb from the telomere on the right arm of chromosome XV (as in Fig 6A but with the localization of the NcoI/AatII restriction sites). Probe used in B is indicated by a solid black line. B In vivo ChEC with Yku70-MN on the left and GBD-MN on the right analyzed on locus depicted in A. Same genomic DNA that used in Fig 6B was digested with NcoI and AatII. The Southern blot was hybridized with a MRM1 specific probe. Time of MNase induction by the addition of Ca2+ is indicated on top of the gel. Ca2+ induced cutting is indicated by a * for Yku70-MN. C Lane profile analysis of the 15 min lanes in B and as in Fig 6C. D Schematic drawing of the modified HIS3 locus (as in Fig 6A) analysed in E. E Genomic DNA from an independent ChEC experiment was digested with NsiI. The Southern blot was hybridized with a HIS3 probe, depicted in D as a solid black line. Time of MNase induction by the addition of Ca2+ is indicated on top of the gel. Ca2+ induced cutting is indicated by a * for Yku70-MN. F Cleavage efficiency by Yku70-MN on ITS with two different probes (MRM1 (B) and HIS3 (E). Quantification was done as described in materials and methods. (PDF) [file pgen.1006479.s006.pdf]

**A**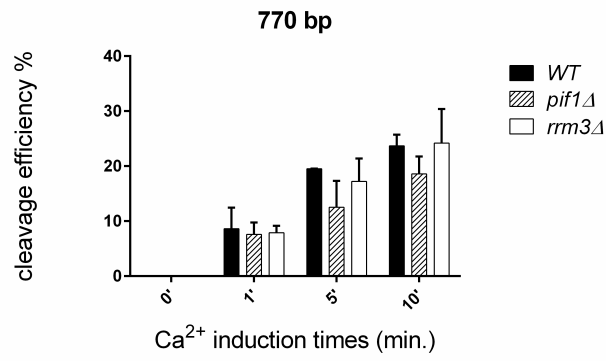**B**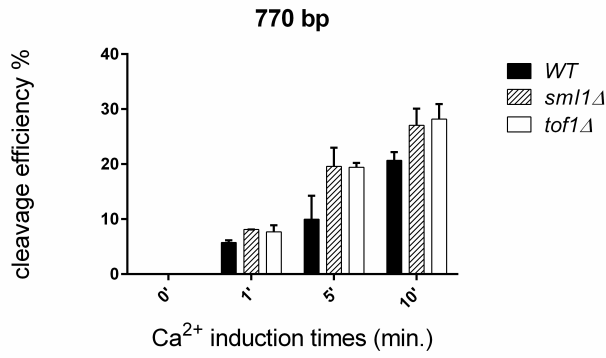**C**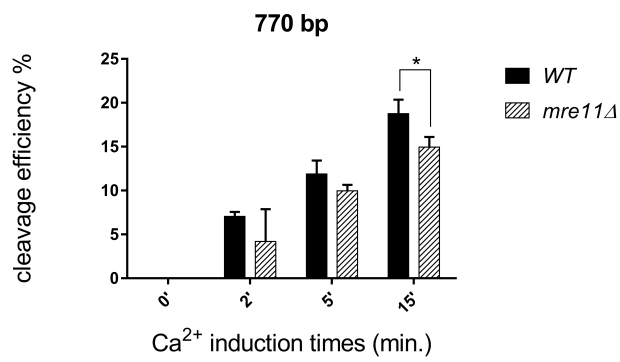**D**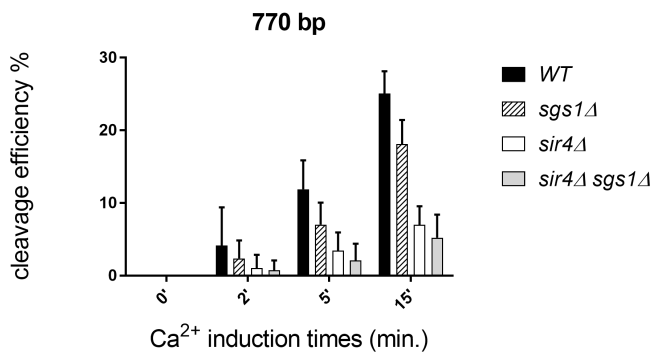S7 Figure ; Larcher *et al.* 2016

Supplement: S7 Fig — A-D Graphs depict the occurrence of cleavage that generated the 770 bp fragment on the Southern blots shown in Fig 7. Significance differences between the WT and mutants strains (P values) were calculated as in Fig 2. Three independent biological replicas were performed for pif1Δ, rrm3Δ, mre11Δ and sgs1Δ alleles and two for sml1Δ and tof1Δ, as Fig 7. P values for the data in A and B indicate no significant differences. (PDF) [file pgen.1006479.s007.pdf]
