# Supplementary material for: Ku Binding on Telomeres Occurs at Sites Distal from the Physical Chromosome Ends
Source: PLoS Genet. 2016 Dec 8;12(12):e1006479. doi: 10.1371/journal.pgen.1006479 (PMC5145143; doi:10.1371/journal.pgen.1006479)
Supplement: S1 Table — (DOCX) [file pgen.1006479.s008.docx]

**S1 Table: Yeast strains used**

| **Strains** | **Characteristics** | **Reference** |
| --- | --- | --- |
| EPY007 | *Mat alpha leu2-2, 112 his3-11,15 trp1-1 MN-L1-Rap1 ura3-1 ade2-2 can1-100 (L1:linker1)* | This study |
| MVY221 | *Mat A Yku70-MN::TRP1 ade2-1 ura3-1 his3-11,15 leu2-3,112 trp1-1 can1-100* | This study |
| MVY60 | *Mat A/alpha, UBR1/ubr1D::GAL1,10-UBR1-HA:HIS3 ade2-1/ade2-1 ura3-1/ura3-1 his3-11,15/his3-11,15 trp1-1 leu2-3,112/leu2-3,112*  *can1-100/can1-100* | This study |
| W303 | *Mat A/alpha ade2-1/ade2-1 ura3-1/ura3-1 his3-11,15/his3-11,15 trp1-1/trp1-1 leu2-3,112/leu2-3,112 can1-100/can1-100* | [[1](#_ENREF_1)] |
| W3749-1a | *Mat A ade2-1 ura3-1 his3-11,15 trp1-1 leu2-3,112 can1-100 bar1∆::LEU2* | [[2](#_ENREF_2)] |
| MVL013 | MVY221 + *bar1Δ::natMX4* | This study |
| MVL022 | MVL013 + *leu2::LEU2-GAL10-FLP1 adh4::FRT-URA3-FRT-TEL [cir°]* | This study |
| MVL023 | MVL013 + *leu2::LEU2-GAL10-FLP1 adh4::FRT-URA3-TEL270-FRT-TEL [cir°]* | This study |
| MVL047 | MVL013 + *sir4Δ::kanMX4* | This study |
| MVL048 | MVL013 + *sir4Δ::kanMX4* | This study |
| MVL052 | MVL013 + *yku80Δ::kanMX4* | This study |
| MVL010 | MVL013 + *sir2Δ::kanMX4* | This study |
| MVL054 | EPY007 + *sir4Δ::kanMX4* | This study |
| MVL030 | MVL013 *+ pif1Δ::kanMX4* | This study |
| MVL031 | MVL013 *+ rrm3Δ::kanMX4* | This study |
| MVL032 | MVL013 *+ sml1Δ::kanMX4* | This study |
| MVL033 | MVL013 *+ tof1Δ::kanMX4* | This study |
| MVL063 | MVL013 *+ mre11Δ::HIS3* | This study |
| MLY30 | *Mat A bar1Δ::HIS3 ade2del::hisG his3del200 leu2del0 lys2del0*  *met15del0 trp1del63 ura3del0* | [[3](#_ENREF_3)] |
| IDY80-1 | MLY30 + *yku80Δ::LEU2* | This study |
| IDY82-9 | MLY30 + *yku80Δ::LEU2 sir4Δ::natMX4* | This study |
| EPY027 | MVL023 + *sir4Δ::kanMX4* | This study |
| EPY028 | MVL022 + *sir4Δ::kanMX4* | This study |
| EPY031 | W303 + *sir4Δ::kanMX4* | This study |
| EPY050 | MVL013 + *sgs1Δ::URA3* | This study |
| EPY052 | MVL047 + *sgs1Δ::URA3* | This study |
| EPY054 | W303 + *his3::empty HIS3* | This study |
| EPY056 | W303 + *his3::TEL_256_ HIS3* | This study |
| EPY058 | EPY031 + *his3::empty HIS3* | This study |
| EPY059 | EPY031 + *his3::TEL_256_ HIS3* | This study |
| EPY061 | MVL013 + *his3::empty HIS3* | This study |
| EPY063 | MVL013 + *his3::TEL_256_ HIS3* | This study |
| EPY064 | MVL047 + *his3::empty HIS3* | This study |
| EPY066 | MVL047 + *his3::TEL_256_ HIS3* | This study |

1. Ronne H, Rothstein R. Mitotic sectored colonies: evidence of heteroduplex DNA formation during direct repeat recombination. Proc Natl Acad Sci U S A. 1988 Apr;85(8):2696-700. PubMed PMID: 3282237. Pubmed Central PMCID: 280065.

2. Lisby M, Barlow JH, Burgess RC, Rothstein R. Choreography of the DNA Damage ResponseSpatiotemporal Relationships among Checkpoint and Repair Proteins. Cell. 2004;118(6):699-713.

3. Larrivee M, LeBel C, Wellinger RJ. The generation of proper constitutive G-tails on yeast telomeres is dependent on the MRX complex. Genes Dev. 2004 Jun 15;18(12):1391-6. PubMed PMID: 15198981.
