# Supplementary material for: Ku Binding on Telomeres Occurs at Sites Distal from the Physical Chromosome Ends
Source: PLoS Genet. 2016 Dec 8;12(12):e1006479. doi: 10.1371/journal.pgen.1006479 (PMC5145143; doi:10.1371/journal.pgen.1006479)
Supplement: S2 Table — (DOCX) [file pgen.1006479.s009.docx]

**S2 Table: Oligonucleotides used**

| **Name** | **Sequence** | **Reference** |
| --- | --- | --- |
| Rap1 promo XhoI For | 5'-ACCGCTCGAGAAGGTTTGGACACAGCATGT-3' | This study |
| Rap1 promo ClaI Rev | 5'-CCGATCGATATTGAGATAATCTGTACGCA-3' | This study |
| Rap1 500 bp ClaI For | 5'-CCGATCGATATGTCTAGTCCAGATGATTT-3' | This study |
| Rap1 500 bp EcorRI Rev | 5'-CCGGAATTCTCTCTTGGATTGGAGTCTAG-3' | This study |
| MN ClaI For | 5'-ACGGTTATCGATGGCAACTTCAACTAAAAAATTACATAAAGAACC-3' | This study |
| MNL1 R | 5'-CCGATCGATAGCATCTCTAGCTTGATCAACTTGACCTGAATCAGCGTTGTCTT-3' | This study |
| F2-Hdf1 | 5'-AAAAGAAGAAAAGAAGCCCTTTGATAAAAAGCCGAAATTCAATATACGGATCCCCGGGTTAATTAATTAA-3' | This study |
| R1-Hdf1 | 5'-ATTGTGTGTAACGTTATAGATATGAAGGATTTCAA  TCGTCTTTAGAATTCGAGCTCGTTAAAC-3' | This study |
| For the q-PCR |  |  |
| CLN2F1 | 5'-CGCACTTTACCCTGAAATGCG-3' | This study |
| CLN2R2 | 5'-TCCGCATGCGGAAACATAACGG-3' | This study |
| T12L-ITR For2 | 5'-CGGCCTTGTCTAACACCATCC-3' | This study |
| T12L-ITR Rev3 | 5'-CGGGTAAGGTGTGACAGCG-3' | This study |
| HO-CF | 5’-TGAGAGCCTTCAACCCAGTCAG-3’ | This study |
| HO-CR | 5’-GAAGACAGTCATAAGTGCGGCG-3’ | This study |
