# Supplementary material for: Ku Binding on Telomeres Occurs at Sites Distal from the Physical Chromosome Ends
Source: PLoS Genet. 2016 Dec 8;12(12):e1006479. doi: 10.1371/journal.pgen.1006479 (PMC5145143; doi:10.1371/journal.pgen.1006479)
Supplement: S3 Table — (DOCX) [file pgen.1006479.s010.docx]

**S3 Table: Plasmids used**

| **Name** | **Description** | **Reference** |
| --- | --- | --- |
| pRS306-MN-Rap1 | *URA3, RAP1 promoter, MN, first 500 bp of Rap1* | This study |
| pFA6a-MN-TRP1 | *TRP1, CEN, MN* | [[1](#_ENREF_1)] |
| pSH62-EBD | *HIS3, CEN, Gal1-Cre-EBD* | [[2](#_ENREF_2)] |
| pRS314-Cre-EBD | *TRP, CEN, Gal1-Cre-EBD* | This study |
| pRSE | *TRP, CEN, Gal1-GBD-MN* | This study |
| pFV17 | *LEU2, TET, Gal10-FLP* | [[3](#_ENREF_3)] |
| sp225 | *URA3, ADH4, FRT* | [[4](#_ENREF_4)] |
| sp229 | *URA3, ADH4, FRT, Tel270* | [[4](#_ENREF_4)] |
| pJP7 | *TRP1, CEN, YKU80-4Gly-2Myc-10HIS* | [[5](#_ENREF_5)] |
| pJP7c | pJP7 *in which a point mutation is corrected* | This study |
| pJP7c-L140A | As pJP7c*,* but *yku80L140A-4Gly-2Myc-10HIS* | This study |
| pJP12 | As pJP7c*,* but *yku80Δ36-4Gly-2Myc-10HIS* | [[5](#_ENREF_5)] |
| pML7c-2 | As pJP7c, marker changed to *HIS3* | This study |
| pML7c-14 | As pJP7c-L140A*,* marker changed to *HIS3* | This study |
| YRpRW40 | *LEU2, URA3, ARS, KANMX4, HO cut site, telo tracts* | [[6](#_ENREF_6)] |
| YRpRW41 | *LEU2, URA3, ARS, KANMX4, HO cut site, telo tracts* | [[6](#_ENREF_6)] |
| YRpRW40-2 | As YRpRW40*, re-established telo tract sizes* | This study |
| pEP19A | *HIS3, TEL_256_* | This study |
| pEP22B | *TRP1, CEN, YKU80-RS-RS-2Myc-10HIS, Gal1-RecR* | This study |
| pEP24C | *TRP1, CEN, YKU80L140A-RS-RS-2Myc-10HIS, Gal1-RecR* | This study |
| YCpHOCUT4 | *URA3, CEN, Gal1-HO, HO Cut site* | [[7](#_ENREF_7)] |

1. Schmid M, Durussel T, Laemmli UK. ChIC and ChECGenomic Mapping of Chromatin Proteins. Molecular Cell. 2004;16(1):147-57.

2. Cheng TH, Chang CR, Joy P, Yablok S, Gartenberg MR. Controlling gene expression in yeast by inducible site-specific recombination. Nucleic Acids Research. 2000 Dec 15;28(24):E108. PubMed PMID: 11121495. Pubmed Central PMCID: 115252. Epub 2000/01/11. eng.

3. Volkert FC, Broach JR. Site-specific recombination promotes plasmid amplification in yeast. Cell. 1986 Aug 15;46(4):541-50. PubMed PMID: 3524855.

4. Marcand S, Brevet V, Gilson E. Progressive cis-inhibition of telomerase upon telomere elongation. Embo J. 1999;18(12):3509-19.

5. Pfingsten JS, Goodrich KJ, Taabazuing C, Ouenzar F, Chartrand P, Cech TR. Mutually exclusive binding of telomerase RNA and DNA by Ku alters telomerase recruitment model. Cell. 2012 Mar 2;148(5):922-32. PubMed PMID: 22365814. Pubmed Central PMCID: 3327133. Epub 2012/03/01. eng.

6. Dionne I, Wellinger RJ. Processing of telomeric DNA ends requires the passage of a replication fork. Nucleic Acids Res. 1998;26(23):5365-71.

7. Raghuraman MK, Brewer BJ, Fangman WL. Activation of a yeast replication origin near a double-stranded DNA break. Genes Dev. 1994;8(5):554-62.
